# Comparison of logistic regression with machine learning methods for the prediction of fetal growth abnormalities: a retrospective cohort study

**DOI:** 10.1186/s12884-018-1971-2

**Published:** 2018-08-15

**Authors:** Stefan Kuhle, Bryan Maguire, Hongqun Zhang, David Hamilton, Alexander C. Allen, K. S. Joseph, Victoria M. Allen

**Affiliations:** 1 Perinatal Epidemiology Research Unit, Departments of Obstetrics & Gynaecology and Pediatrics, Dalhousie University, Halifax, NS Canada; 2 Department of Mathematics & Statistics, Dalhousie University, Halifax, NS Canada; 3 Department of Obstetrics & Gynaecology and School of Population & Public Health, University of British Columbia, Vancouver, BC Canada; 4 Department of Obstetrics & Gynaecology, Dalhousie University, Halifax, NS Canada

**Keywords:** Pregnancy, Infant, Prediction, Birth weight, Fetal growth restriction, Fetal macrosomia

## Abstract

**Background:**

While there is increasing interest in identifying pregnancies at risk for adverse outcome, existing prediction models have not adequately assessed population-based risks, and have been based on conventional regression methods. The objective of the current study was to identify predictors of fetal growth abnormalities using logistic regression and machine learning methods, and compare diagnostic properties in a population-based sample of infants.

**Methods:**

Data for 30,705 singleton infants born between 2009 and 2014 to mothers resident in Nova Scotia, Canada was obtained from the Nova Scotia Atlee Perinatal Database. Primary outcomes were small (SGA) and large for gestational age (LGA). Maternal characteristics pre-pregnancy and at 26 weeks were studied as predictors. Logistic regression and select machine learning methods were used to build the models, stratified by parity. Area under the curve was used to compare the models; relative importance of predictors was compared qualitatively.

**Results:**

7.9% and 13.5% of infants were SGA and LGA, respectively; 48.6% of births were to primiparous women and 51.4% were to multiparous women. Prediction of SGA and LGA was poor to fair (area under the curve 60–75%) and improved with increasing parity and pregnancy information. Smoking, previous low birthweight infant, and gestational weight gain were important predictors for SGA; pre-pregnancy body mass index, gestational weight gain, and previous macrosomic infant were the strongest predictors for LGA.

**Conclusions:**

The machine learning methods used in this study did not offer any advantage over logistic regression in the prediction of fetal growth abnormalities. Prediction accuracy for SGA and LGA based on maternal information is poor for primiparous women and fair for multiparous women.

**Electronic supplementary material:**

The online version of this article (10.1186/s12884-018-1971-2) contains supplementary material, which is available to authorized users.

## Background

Normal fetal growth is critical for both short- and long-term health outcomes in neonates [1]. Infants at both tails of the birthweight distribution are responsible for the majority of morbidity and health care costs in neonates born at term [2–4]. Being born small for gestational age (SGA) is associated with seizures, respiratory distress, hypoglycaemia, hyperbilirubinaemia, polycythaemia, thrombocytopenia, and necrotizing enterocolitis [1]. The perinatal morbidity associated with large-for-gestational age (LGA) infants is related to prolonged and complicated labour due to physical size and includes birth injury, the need for operative vaginal delivery or caesarean section, asphyxia, and meconium aspiration [1]. Other postnatal problems commonly seen in LGA infants are hypoglycaemia, polycythaemia, hyperbilirubinaemia, and respiratory distress. SGA and LGA infants on average have higher health care utilization, including during the delivery admission and for readmissions within two weeks of delivery, than neonates born appropriate for gestational age (AGA) [2].

The timely identification of pregnancies at risk for adverse obstetrical and perinatal outcomes may be beneficial to the women, the infants, and the health care system. While there is increasing clinical and administrative interest in predicting which pregnancy will end with an adverse outcome, few studies have used appropriate methods to assess population-based, and gestational age-dependent risks for adverse obstetrical and perinatal outcomes. Studying predictive factors and developing prognostic models to determine the probability of specific obstetrical and perinatal outcomes has important implications for preconception counselling, antenatal assessment, intrapartum care and management in the postpartum period, and for future reproductive health. Prediction models may also help policy makers to predict population changes in outcome frequency following changes in underlying risk factors.

The prediction of adverse obstetrical and perinatal outcomes has been typically based on conventional regression models and has not benefitted from newer techniques such as machine learning. Machine learning describes a methodology for developing algorithms that learn from existing data to make predictions on new data. In contrast to logistic regression, machine learning methods such as random forest, boosting, or neural networks have no underlying distributional assumptions, can handle complex relationships between predictors and the outcome, as well as a larger number of predictors, and require no model specification [5–7]. Machine learning has become an integral component of many technologies used in everyday life (from credit card fraud detection to movie recommendations), but its use in clinical and population health research has been relatively limited. Reasons for this delayed uptake include the limited availability of such methods in mainstream statistical software packages, the specialized knowledge that is required for their use, and clinician preference for easily understood approaches over “black box” prediction methods.

The objective of the present study was to use conventional regression models and various machine learning methods to identify predictors of fetal growth abnormalities and compare their diagnostic properties (sensitivity, specificity, positive and negative predictive value, accuracy, and area under the curve [AUC]) in a large population-based sample of pregnant women from the Canadian province of Nova Scotia.

## Methods

A retrospective cohort of infants born between January 1, 2009 and December 31, 2014 to mothers resident in the Canadian province of Nova Scotia (population in 2016: 923,598) was identified using the Nova Scotia Atlee Perinatal Database (NSAPD). All singleton infants with complete information on the predictors and outcome were included in the study.

### Data source

The NSAPD contains extensive information on routine demographic variables, medical conditions, reproductive history, delivery events, and neonatal outcomes for each birth in the province. Data are entered into the NSAPD by trained coders from standardized clinical forms. Nova Scotia uses a standard Prenatal Record in addition to forms completed at the time of the hospital delivery admission to document prenatal care and information relevant to care and medical research. Its use throughout pregnancy maximizes the completeness and accuracy of information on items like demographics, health-related behaviours, and pregnancy history. The NSAPD is administered by the Reproductive Care Program of Nova Scotia, which also maintains the coding system, and ensures the quality, integrity and security of the data. Periodic abstraction and validation studies form an ongoing data quality assurance program and have shown that the data are accurate and reliable [8].

### Outcomes

The primary outcome was birthweight for gestational age category. Infants were categorized as SGA (< 10th percentile of birthweight for gestational age and sex), LGA (> 90th percentile of birthweight for gestational age and sex), or AGA (10th to 90th percentile of birthweight for gestational age and sex) relative to the Canadian reference population published by Kramer et al. [9]. Secondary outcomes included birthweight for gestational age z-score and SGA defined as birthweight <3rd percentile for gestational age and sex. Gestational age was determined based on an algorithm that used information from the last menstrual period, fetal ultrasound (where available), and the neonatal physical examination [8].

### Predictors

We used demographic and clinical characteristics recorded in the NSAPD that were available as predictors at two time points: pre-pregnancy and at 26 weeks gestation (Additional file 1: Table S1). Area-level income quintile was calculated from the adjusted annual income based on census data averaged over all households in a census dissemination area. Area of residence was determined from the mother’s postal code at the time of pregnancy. Any smoking at 20 weeks or during the labour admission was used as a proxy for smoking at 26 weeks. Pre-pregnancy body mass index (BMI) was based on height and weight information collected by self-report at the first prenatal visit. Gestational weight gain at 26 weeks was estimated as

$$ 2+\left( 13\;x\;\left( Delivery\kern0.17em weight\hbox{-} Pre\hbox{-} pregnancy\kern0.17em weight\hbox{-} 2\right)/\left( Gestational\; age\; at\; birth\hbox{-} 13\right)\right) $$

assuming 2 kg gain in the first trimester (13 weeks) and a steady rate of weight gain thereafter [10].

### Statistical analysis

We developed prediction models for SGA (versus not SGA) and LGA (versus not LGA) on predictors available before pregnancy and at 26 weeks gestation, respectively, using multiple logistic regression, as well as select machine learning methods: elastic net, classification trees, random forest, gradient boosting, and neural networks. Separate models were developed for primiparous and multiparous women. Since machine learning algorithms do not perform well in the presence of imbalance of the predicted classes (e.g., 10% SGA vs. 90% non-SGA), the minority class (SGA or LGA) was upweighted prior to model development to achieve a 1:1 ratio of classes [11]. Observations with missing predictors were excluded from the analysis. Models were implemented in R/RStudio [12, 13] with the *caret* package [14].

Akaike Information Criterion-based model selection was used to build the **logistic regression** models. The Akaike Information Criterion is a method of model selection that deals with the trade-off between the goodness of fit of the model and the complexity of the model. We used the *MASS* package [15] to develop the logistic regression models. **Elastic net** is a penalized regression method that shrinks coefficients toward zero [16]. Covariates that do not significantly improve the fit of the model are shrunk until they are forced out of the model entirely. This method is useful for reducing the number of covariates included in the model and dealing with groups of correlated covariates. We used the *glmnet* package [17] to build the Elastic Net models. **Classification trees** are developed via a nonparametric recursive partitioning method whereby the sample is successively divided by binary splits. At each successive step, splits (node) are made at the cutpoint which maximizes the discrimination between those who develop the outcome (cases) and those who do not (non-cases). Each node is a decision that creates a new “branch” in the “tree”. Once no more divisions can be made, a terminal node is reached and a prediction is made. We used the *rpart* package [18] to build the trees. A **random forest** is a collection of decision trees, each constructed in a bootstrapped sample and from a random subset of the possible predictors at each node to make a prediction. The mode of these predictions is the final prediction of the model. Random forests are used to reduce variance and overfitting associated with decision trees [5]. We used the *randomForest* package [19] to develop the random forest models. **Gradient boosting** repeatedly applies a classification algorithm to a weighted version of the training data, more heavily weighting those observations that previous iterations have frequently misclassified [7]. A vote of the predictions for each iteration is used to create a final prediction. This method attempts to give more attention to those data points that are difficult to classify. We used the *gbm* package [20] to build the gradient boosting models. **Neural Networks** are composed of layers of many simple predictive functions that are connected via weights. These weights are determined by repeatedly comparing the output of the network to the training data set and adjusting. This collection of predictive functions is often compared to the way neurons of the brain are connected to make complex decisions. We used the *nnet* package [21] to build the neural network models.

Data was randomly assigned to a training (80%) and a testing (20%) data set. Ten-fold cross validation in the training data was used to develop the prediction models. The training parameter grids and parameters used for each method are shown in Additional file 2: Table S2; the AUC in the training data is shown in Additional file 3: Table S3. The AUC and accuracy of predictions in the test data were used to compare the models generated by the different methods. The relative importance of predictors was compared qualitatively between methods using variable importance plots.

### Ethics

The Reproductive Care Program of Nova Scotia and the Research Ethics Board of the IWK Health Centre (File # 1015714) provided data access approval and ethics approval, respectively. The need for informed consent for this database-based study was waived as per the Nova Scotia Personal Health Information Act. All procedures performed were in accordance with the ethical standards of the institutional research committee and with the *Tri-Council Policy Statement: Ethical Conduct for Research Involving Humans, December 2014*.

## Results

Over the study period from 2009 to 2014, there were 49,604 pregnancies in women residents of Nova Scotia that resulted in a singleton live birth after 26 weeks gestation; for 30,705 pregnancies, complete information on all variables was available, and these pregnancies were included in the study sample. Most exclusions (*n* = 13,161) were due to missing pre-pregnancy weight, pregnancy weight, or both.

7.9% and 13.5% of births were SGA and LGA, respectively; 48.6% of pregnancies were to primiparous women and 51.4% were to multiparous women. The predictors are summarized by birthweight for gestational age category in Table 1. The most pronounced differences compared to AGA infants were seen for smoking (higher in SGA), pre-pregnancy BMI (higher in LGA), and gestational weight gain (highest in LGA and lowest in SGA groups).

**Table 1:** Sample characteristics by parity and birthweight for gestational age category (*N* = 30,705)

Predictors	Primiparae			Multiparae		
	AGA	SGA	LGA	AGA	SGA	LGA
Sociodemographics						
Maternal age [years]	27.2 (5.7)	26.9 (6.0)	27.3 (5.5)	30.3 (5.2)	29.8 (5.5)	31.1 (4.9)
Common-law/married	66%	63%	67%	79%	68%	84%
Area-level income quintiles	18/22/23/22/15%	22/21/24/20/13%	16/23/22/24/15%	17/21/23/22/17%	24/22/22/18/14%	17/19/24/22/18%
Urban residence	66%	63%	67%	79%	68%	84%
Pregnancy risk factors						
Smoking before pregnancy	25%	37%	20%	24%	47%	15%
Pre-pregnancy BMI [m/kg^2^]	25.5 (6.1)	24.9 (6.4)	27.7 (6.8)	26.4 (6.4)	24.9 (6.0)	28.8 (7.2)
Pre-existing hypertension	1%	2%	2%	1%	2%	2%
Pre-existing diabetes	1%	1%	2%	1%	1%	3%
Past pregnancy history						
Previous gestational diabetes	–	–	–	3%	2%	5%
Previous child with BW < 2500 g	–	–	–	7%	19%	3%
Previous child with BW > 4080 g	–	–	–	9%	3%	31%
Previous caesarean section	–	–	–	24%	23%	29%
Previous preterm delivery < 29 wks	–	–	–	1%	1%	1%
Previous preterm delivery 29–32 wks	–	–	–	1%	2%	1%
Previous preterm delivery 33–36 wks	–	–	–	5%	7%	4%
Previous death of neonate ≥500 g	–	–	–	1%	1%	0%
Current pregnancy						
Fetal male sex	51%	54%	52%	51%	52%	50%
Weight gain at 26 wks [kg]	8.9 (3.3)	7.9 (3.1)	10.3 (3.8)	7.8 (3.2)	6.7 (3.2)	8.8 (3.5)
Smoking during pregnancy	15%	27%	9%	19%	41%	10%
Substance use in pregnancy	3%	5%	2%	2%	4%	1%
Gestational diabetes	4%	6%	8%	5%	5%	10%
Pregnancy-induced hypertension	2%	5%	2%	1%	2%	1%
Psychiatric disorder	11%	13%	11%	12%	14%	10%

Numbers are presented as mean (standard deviation) or proportions as applicable

Abbreviations: *AGA* appropriate for gestational age, *BMI* body mass index, *BW* birthweight, *LGA* large for gestational age, *Pre-P* pre-pregnancy, *SGA* small for gestational age, *wks* weeks

Tables 2 and 3 show the AUC, accuracy, and the most important predictors for SGA and LGA models, respectively. For both SGA and LGA, the predictions were poor (AUC 0.6–0.7) for primiparous women and fair (AUC 0.7–0.8) for multiparous women, irrespective of the method used. Within time point and parity strata, the differences in AUC between the methods were negligible (confidence intervals for the AUC estimates were approximately ±0.03). The predictions improved in the order Primipara/Pre-Pregnancy, Primipara/26 weeks, Multipara/Pre-Pregnancy, Multipara/26 weeks. The ROC curves for each model can be found in Additional file 4: Figure S1, Additional file 5: Figure S2, Additional file 6: Figure S3, Additional file 7: Figure S4, Additional file 8: Figure S5, Additional file 9: Figure S6, Additional file 10: Figure S7 and Additional file 11: Figure S8.

**Table 2:** Area under the curve, accuracy, and the three most important predictors for the prediction of small for gestational age (SGA) birth using logistic regression and five machine learning methods pre-pregnancy and at 26 weeks in primiparous and multiparous women

	Pre-pregnancy						26 weeks					
	LR	EN	CT	RF	GB	NN	LR	EN	CT	RF	GB	NN
SGA - Primiparae												
Area under the curve	0.592	0.598	0.569	0.601	0.609	0.600	0.662	0.661	0.627	0.650	0.665	0.660
Accuracy	0.839	0.845	0.815	0.841	0.851	0.841	0.847	0.849	0.829	0.844	0.846	0.849
Maternal age		●	●	●	●							
Area-level income quintile						●						
Pre-pregnancy smoking	●	●	●	●	●	●	●	●				
Pre-pregnancy BMI	●		●	●	●				●	●	●	
Pre-existing hypertension		●				●		●				●
Gravidity	●									●		
Weight gain at 26 wks							●		●	●	●	
Smoking in pregnancy							●		●		●	●
Pregnancy-induced hypertension								●				●
SGA – Multiparae												
Area under the curve	0.741	0.744	0.711	0.715	0.728	0.741	0.771	0.771	0.713	0.745	0.766	0.772
Accuracy	0.905	0.903	0.916	0.897	0.902	0.906	0.912	0.912	0.801	0.903	0.911	0.914
Pre-pregnancy smoking	●	●		●	●		●	●				
Pre-pregnancy BMI	●		●	●	●				●	●	●	
Pre-existing hypertension		●										
Previous LBW infant	●	●	●	●		●		●		●		●
Previous infant > 4080 g			●		●	●			●			●
Previous preterm delivery < 29 wks						●						
Weight gain at 26 wks							●		●	●	●	
Smoking in pregnancy							●				●	
Pregnancy-induced hypertension								●				●

Abbreviations: *BMI* body mass index, *CT* classification tree, *EN* elastic net, *GB* gradient boosting, *LBW* low birth weight, *LR* logistic regression, *NN* neural network, *RF* random forest, *wks* weeks

**Table 3:** Area under the curve, accuracy, and the three most important predictors for the prediction of large for gestational age (LGA) birth using logistic regression and five machine learning methods pre-pregnancy and at 26 weeks in nulliparous and multiparous women

	Pre-pregnancy						26 weeks					
	LR	EN	CT	RF	GB	NN	LR	EN	CART	RF	GB	NN
LGA - Primiparae												
Area under the curve	0.592	0.587	0.563	0.576	0.587	0.594	0.702	0.705	0.675	0.673	0.697	0.705
Accuracy	0.826	0.827	0.800	0.824	0.832	0.827	0.843	0.834	0.780	0.834	0.839	0.842
Maternal age	●	●	●									
Common-law/married	●											
Pre-pregnancy smoking			●	●	●	●						
Pre-pregnancy BMI	●	●	●	●	●	●	●	●	●		●	
Pre-existing diabetes		●		●	●	●		●		●		●
Weight gain at 26 wks							●	●	●	●	●	
Smoking in pregnancy									●		●	●
Pregnancy-induced hypertension										●		●
Gestational diabetes							●					
LGA - Multiparae												
Area under the curve	0.700	0.700	0.659	0.692	0.704	0.700	0.745	0.748	0.718	0.728	0.748	0.746
Accuracy	0.807	0.806	0.817	0.795	0.804	0.807	0.813	0.809	0.794	0.799	0.805	0.812
Maternal age	●											
Pre-pregnancy smoking			●		●							
Pre-pregnancy BMI	●	●	●	●	●		●			●	●	
Pre-existing diabetes		●		●		●		●				●
Previous LBW infant												
Previous infant > 4080 g	●	●	●	●	●	●	●	●	●	●	●	●
Previous death of neonate ≥500 g						●						●
Weight gain at 26 wks							●	●	●	●	●	
Smoking in pregnancy									●			

Abbreviations: *BMI* body mass index, *CT* classification tree, *EN* elastic net, *GB* Gradient boosting, *LBW* low birth weight, *LR* logistic regression, *NN* neural network, *RF* random forest, *wks* weeks

The most important predictors for each time point and stratum were similar between methods. Smoking, a previous LBW infant, and gestational weight gain were consistently identified as strong predictors of SGA, while pre-pregnancy BMI, gestational weight gain, and a previous infant > 4080 g were the strongest predictors of LGA across all methods. The addition of information on the size of a previous infant (either < 2500 g or > 4080 g) provided the greatest gain in information when going from primiparous to multiparous models. Weight gain at 26 weeks in turn was an important predictor when going from pre-pregnancy to 26 weeks.

In a secondary analysis, we also developed prediction models for SGA defined as birthweight for gestational age and sex <3rd percentile, as well as for a continuous version of the outcome (birthweight for gestational age z-score), but these models did not offer any advantage over the models for the primary outcomes.

## Discussion

We attempted to identify predictors of fetal growth abnormalities using population-based data with logistic regression and selected machine learning and compare their diagnostic properties. Rates of SGA and LGA live births observed in this study were consistent with nationally reported rates [22]. We found that the predictions were poor to fair for both SGA and LGA. Predictions were best for multiparous women at 26 weeks and poorest for primiparous women pre-pregnancy. None of the prediction methods offered any advantages over the others in terms of AUC. Smoking, a previous LBW infant, and gestational weight gain were consistently identified as strong predictors for SGA, while pre-pregnancy BMI, gestational weight gain, and a previous infant > 4080 g were the strongest predictors for LGA.

Most published models are based on fetal ultrasound measurements at some point during pregnancy or include biochemical markers. The current study predicted SGA and LGA births based on readily available clinical characteristics that may be used in situations where imaging or laboratory testing is not available or has not been utilized. We considered pre-pregnancy and late 2nd trimester factors available in the NSAPD to evaluate their predictive ability prior to the third trimester, when there is an increased risk of obstetrical complications associated with SGA and LGA that alter obstetrical management decisions. Prediction models in the literature that are based on maternal characteristics have an AUC of about 0.70 for SGA and LGA [23–25]. Ultrasound evaluation of fetal size in the third trimester is superior in terms of the AUC (0.80–0.90 for SGA and LGA) [26–29] but repeated evaluations of fetal growth have not been shown to provide additional information compared to a single measurement before before 33 weeks gestation [27]. The use of ultrasound biometry and corresponding growth curves in the prediction of estimated fetal size are limited by the populations from which they were derived, as well as by maternal body habitus and gestational age; as a result, fetal biometry has an error in the range of 10% to 15%, especially at the two extremes of size [30]. Integrating maternal characteristics with ultrasound information and maternal serum biomarkers has been proposed in the clarification of risks for SGA and LGA [31]. Models incorporating first trimester ultrasound parameters, biochemical indices, and maternal characteristics had AUCs up to 0.73 for both SGA [23, 32, 33] and LGA [23, 25, 34]. Our approach of stratifying by parity and timing allowed for more flexibility in the selection of predictors for the separate models. The diagnostic properties of the models for multiparous women are comparable to those of the models integrating first trimester clinical, biochemical, and imaging information, but predictions from the models for primiparous women were considerably weaker.

Previous studies on the prediction of SGA and LGA have commonly used logistic regression to develop models. Our study was the first of which we are aware that used and compared machine learning methods in the prediction of fetal growth abnormalities. The advantages of logistic regression models include the comparatively easy implementation, the availability in all standard statistical software packages, and short computation times. However, misspecification of the logistic regression model or violation of its assumptions may result in biased results. By contrast, the machine learning methods used in the current study (with the exception of elastic net) make no distributional assumptions, do not require a priori specification of a model, and can consider complex relationships between the predictors and the outcome. The fact that the machine learning methods used in the current study did not perform better than a conventional logistic regression model indicates that the relationship between predictors and the two outcomes may not be complex, and therefore the strengths of machine learning methods over conventional regression did not play a role. Several studies have compared machine learning methods to conventional logistic regression for prediction for a variety of clinical conditions, and the results regarding the diagnostic properties of the models have shown mixed results [6, 35, 36], underlining that there is no overall “best” method for prediction and that the choice of the optimal method is dependent on the specific setting. Our findings should therefore not discourage the use of machine learning methods in evaluating other areas of clinical obstetrics and gynaecology.

Logistic regression models provide effect estimates (odds ratios) that are easily interpretable, whereas machine learning methods are often considered “black box” methods as they do not readily provide the user with any indication of the importance of individual predictors that are used for the prediction output. Some machine learning methods offer variable importance rankings that order predictors in the model based on the loss of prediction accuracy when they are removed from the model. These rankings can give the user some indication of the relative importance of the predictors. Previous studies using logistic regression models have identified underweight, short stature, inadequate gestational weight, pre-eclampsia, smoking, maternal age under 18 or over 35, primigravidity, and history of a SGA infant as strong risk factors for SGA births [37–39], while maternal obesity, non-smoking, maternal age, high gestational weight gain, and multiparity were identified as strongest predictors of macrosomia [40]. The highest ranked predictors of SGA and LGA based on the variable importance rankings from the machine learning methods used in the present study identified the latter predictors but also highlighted some unusual predictors such as previous preterm birth (SGA, pre-pregnancy multiparity model, neural network) or previous death of a neonate ≥500 g (LGA, pre-pregnancy multipara model, neural network). Owing to the different algorithms used with each method, predictor importance may differ greatly between methods, and a high ranking of a predictor may not necessarily translate into a high odds ratio for the same variable in a conventional regression model.

The strengths of the study are the use of a comprehensive, population-based perinatal database with a broad range of high quality data. Our study was limited by the lack of an external validation, which may have resulted in overly optimistic estimates of the diagnostic properties of the prediction models. In addition, maternal BMI was based on self-reported data, which may result in misclassification of weight status; however, self-reported pre-pregnancy weight has been shown to agree closely with measured weight [41]. Another limitation was the exclusion of a large number of mothers with missing information (*n* = 18,899, 38%), in particular for BMI and gestational weight gain, which may have led to a selection of women with higher BMIs in the analysis sample as they may be more likely to have their weight and height recorded. However, the proportion of SGA and LGA was very similar in the included (SGA: 7.90%; LGA: 13.52%) and excluded infants (SGA: 7.90%; LGA: 13.76%), which does not support the latter hypothesis. Since a certain proportion of SGA and LGA infants may be otherwise healthy, our prediction models may identify some infants without associated morbidity. Future research should examine if the predictors examined in the current study can identify adverse outcomes of SGA and LGA directly; such an assessment was beyond the scope of this study. Despite the broad range of data, we were limited by variables in the NSAPD, and were not able to include information on factors such as racial origin, ultrasound biometry, or maternal serum biomarkers. Lastly, other, more complex machine learning methods (such as Deep Learning or Super Learners) than the ones used in the current study may offer greater prediction accuracy.

## Conclusions

Prediction of fetal growth abnormalities based on sociodemographic and clinical information is of limited value for primiparous women, but prediction accuracy is fair for multiparous women pre-pregnancy and at 26 weeks gestation. The machine learning methods used in the current study did not offer any advantages over conventional logistic regression in the prediction of SGA and LGA status. Smoking, a previous LBW infant, and gestational weight gain were identified by most methods as key predictors for SGA, while pre-pregnancy BMI, gestational weight gain, and a previous infant > 4080 g were key predictors for LGA.

## Additional files

Additional file 1: **Table S1.** Predictors of fetal growth abnormalities and their use in the prediction models. (PDF 71 kb)

Additional file 2: **Table S2.** Training parameter grids and parameters used for five machine learning methods for the prediction of fetal growth abnormalities. (PDF 186 kb)

Additional file 3: **Table S3.** Area under the curve in the training data for logistic regression and five machine learning methods for the prediction of fetal growth abnormalities. (PDF 179 kb)

Additional file 4: **Figure S1.** Receiver operating characteristic curves for the prediction of SGA among primiparous women (pre-pregnancy) using elastic net, classification trees, random forest, gradient boosting, and neural networks. (PDF 176 kb)

Additional file 5: **Figure S2.** Receiver operating characteristic curves for the prediction of SGA among primiparous women (26 weeks) using elastic net, classification trees, random forest, gradient boosting, and neural networks. (PDF 186 kb)

Additional file 6: **Figure S3.** Receiver operating characteristic curves for the prediction of SGA among multiparous women (pre-pregnancy) using elastic net, classification trees, random forest, gradient boosting, and neural networks. (PDF 188 kb)

Additional file 7: **Figure S4.** Receiver operating characteristic curves for the prediction of SGA among multiparous women (26 weeks) using elastic net, classification trees, random forest, gradient boosting, and neural networks. (PDF 189 kb)

Additional file 8: **Figure S5.** Receiver operating characteristic curves for the prediction of LGA among primiparous women (pre-pregnancy) using elastic net, classification trees, random forest, gradient boosting, and neural networks. (PDF 180 kb)

Additional file 9: **Figure S6.** Receiver operating characteristic curves for the prediction of LGA among primiparous women (26 weeks) using elastic net, classification trees, random forest, gradient boosting, and neural networks. (PDF 192 kb)

Additional file 10: **Figure S7.** Receiver operating characteristic curves for the prediction of LGA among multiparous women (pre-pregnancy) using elastic net, classification trees, random forest, gradient boosting, and neural networks. (PDF 200 kb)

Additional file 11: **Figure S8.** Receiver operating characteristic curves for the prediction of LGA among multiparous women (26 weeks) using elastic net, classification trees, random forest, gradient boosting, and neural networks. (PDF 201 kb)

## Abbreviations

AGA: Appropriate for gestational age
AUC: Area under the curve
BMI: Body mass index
LBW: Low birth weight
LGA: Large for gestational age
NSAPD: Nova Scotia Atlee Perinatal Database
SGA: Small for gestational age
