# Supplementary material for: Comparison of logistic regression with machine learning methods for the prediction of fetal growth abnormalities: a retrospective cohort study
Source: BMC Pregnancy Childbirth. 2018 Aug 15;18:333. doi: 10.1186/s12884-018-1971-2 (PMC6094446; doi:10.1186/s12884-018-1971-2)
Supplement: Supplementary file 1 — Table S1. Predictors of fetal growth abnormalities and their use in the prediction models. (PDF 71 kb) [file 12884_2018_1971_MOESM1_ESM.pdf]

Table S1: Predictors of fetal growth abnormalities and their use in the prediction models.

Predictors	Type	Primiparae		Multiparae	
		Pre-Pregnancy	26 weeks	Pre-Pregnancy	26 weeks
**Sociodemographics**					
Maternal age	Categorical	●	●	●	●
Common-law/married	Binary	●	●	●	●
Area-level income quintile	Categorical	●	●	●	●
Area of residence (urban/rural)	Binary	●	●	●	●
**Pregnancy risk factors**					
Smoking before pregnancy	Binary	●	●	●	●
Pre-pregnancy body mass index	Continuous	●	●	●	●
Pre-existing hypertension	Binary	●	●	●	●
Pre-existing diabetes	Binary	●	●	●	●
**Past pregnancy history**					
Gravidity	Discrete	●	●	●	●
Parity	Discrete			●	●
Previous gestational diabetes	Binary			●	●
Previous child with birthweight < 2500g	Binary			●	●
Previous child with birthweight > 4080g	Binary			●	●
Previous caesarean section	Binary			●	●
Previous preterm delivery < 29 weeks	Binary			●	●
Previous preterm delivery 29-32 weeks	Binary			●	●
Previous preterm delivery 33-36 weeks	Binary			●	●
Previous death of a neonate ≥ 500g	Binary			●	●
**Current pregnancy**					
Fetal sex	Binary		●		●
Weight gain in pregnancy at 26 weeks	Continuous		●		●
Smoking during pregnancy	Binary		●		●
Substance use in pregnancy	Binary		●		●
Gestational diabetes	Binary		●		●
Pregnancy-induced hypertension	Binary		●		●
Psychiatric disorder	Binary		●		●
