# Supplementary material for: Comparison of logistic regression with machine learning methods for the prediction of fetal growth abnormalities: a retrospective cohort study
Source: BMC Pregnancy Childbirth. 2018 Aug 15;18:333. doi: 10.1186/s12884-018-1971-2 (PMC6094446; doi:10.1186/s12884-018-1971-2)
Supplement: Supplementary file 2 — Table S2. Training parameter grids and parameters used for five machine learning methods for the prediction of fetal growth abnormalities. (PDF 186 kb) [file 12884_2018_1971_MOESM2_ESM.pdf]

Table S2: Training parameter grids and parameters used for five machine learning methods for the prediction of fetal growth abnormalities.

		SGA				LGA			
		Primiparae		Multiparae		Primiparae		Multiparae	
**Method**	**Training parameter range**	**Pre-pregnancy**	**26 weeks**	**Pre-pregnancy**	**26 weeks**	**Pre-pregnancy**	**26 weeks**	**Pre-pregnancy**	**26 weeks**
EN	alpha: (0:10)/100	0.6	0.5	0.2	0.8	0.2	0.2	0.3	0.5
	lambda: (1:10)/100	0.02	0.01	0.05	0.01	0.07	0.01	0.01	0.02
CT	complexity parameter: (1:10)/1000	0.002	0.002	0.006	0.004	0.002	0.004	0.004	0.002
RF	mtry: 1:6	2	2	2	3	2	2	2	2
	n of trees: 50, 200, 500	200	200	200	200	200	200	500	200
GB	n of trees: 200	200	200	200	200	200	200	200	200
	interaction depth: 1:5	2	1	1	2	2	1	1	2
	shrinkage: 0.1, 0.01	0.01	0.1	0.1	0.1	0.01	0.1	0.1	0.1
	minimum node size: 10, 20	20	10	10	10	20	20	20	10
NN	decay: 0.1, 0.2, 0.5	0.1	0.2	0.1	0.5	0.1	0.2	0.2	0.5
	size: 1:5	1	1	1	1	1	1	1	1

Abbreviations: *CT* classification tree, *EN* elastic net, *GB* gradient boosting, *LGA* large for gestational age, *LR* logistic regression, *mtry* number of variables randomly sampled as candidates at each split, *n* number, *NN* neural network, *RF* random forest, *SGA* small for gestational age
