# Supplementary material for: Comparison of logistic regression with machine learning methods for the prediction of fetal growth abnormalities: a retrospective cohort study
Source: BMC Pregnancy Childbirth. 2018 Aug 15;18:333. doi: 10.1186/s12884-018-1971-2 (PMC6094446; doi:10.1186/s12884-018-1971-2)
Supplement: Supplementary file 3 — Table S3. Area under the curve in the training data for logistic regression and five machine learning methods for the prediction of fetal growth abnormalities. (PDF 179 kb) [file 12884_2018_1971_MOESM3_ESM.pdf]

Table S3: Area under the curve in the training data for logistic regression and five machine learning methods for the prediction of fetal growth abnormalities.

	SGA				LGA			
	Primiparae		Multiparae		Primiparae		Multiparae	
**Method**	**Pre-pregnancy**	**26 weeks**	**Pre-pregnancy**	**26 weeks**	**Pre-pregnancy**	**26 weeks**	**Pre-pregnancy**	**26 weeks**
LR	0.599	0.673	0.714	0.752	0.627	0.704	0.707	0.747
EN	0.601	0.672	0.709	0.749	0.626	0.705	0.707	0.744
CT	0.614	0.705	0.688	0.707	0.641	0.678	0.670	0.729
RF	0.711	0.822	0.811	0.880	0.732	0.855	0.773	0.838
GB	0.613	0.687	0.734	0.769	0.634	0.713	0.714	0.750
NN	0.604	0.674	0.714	0.753	0.629	0.701	0.707	0.747

Abbreviations: *CT* classification tree, *EN* elastic net, *GB* gradient boosting, *LGA* large for gestational age, *LR* logistic regression, *NN* neural network, *RF* random forest, *SGA* small for gestational age
