# Supplementary figures and images for: Comparison of logistic regression with machine learning methods for the prediction of fetal growth abnormalities: a retrospective cohort study
Source: BMC Pregnancy Childbirth. 2018 Aug 15;18:333. doi: 10.1186/s12884-018-1971-2 (PMC6094446; doi:10.1186/s12884-018-1971-2)

**EN**

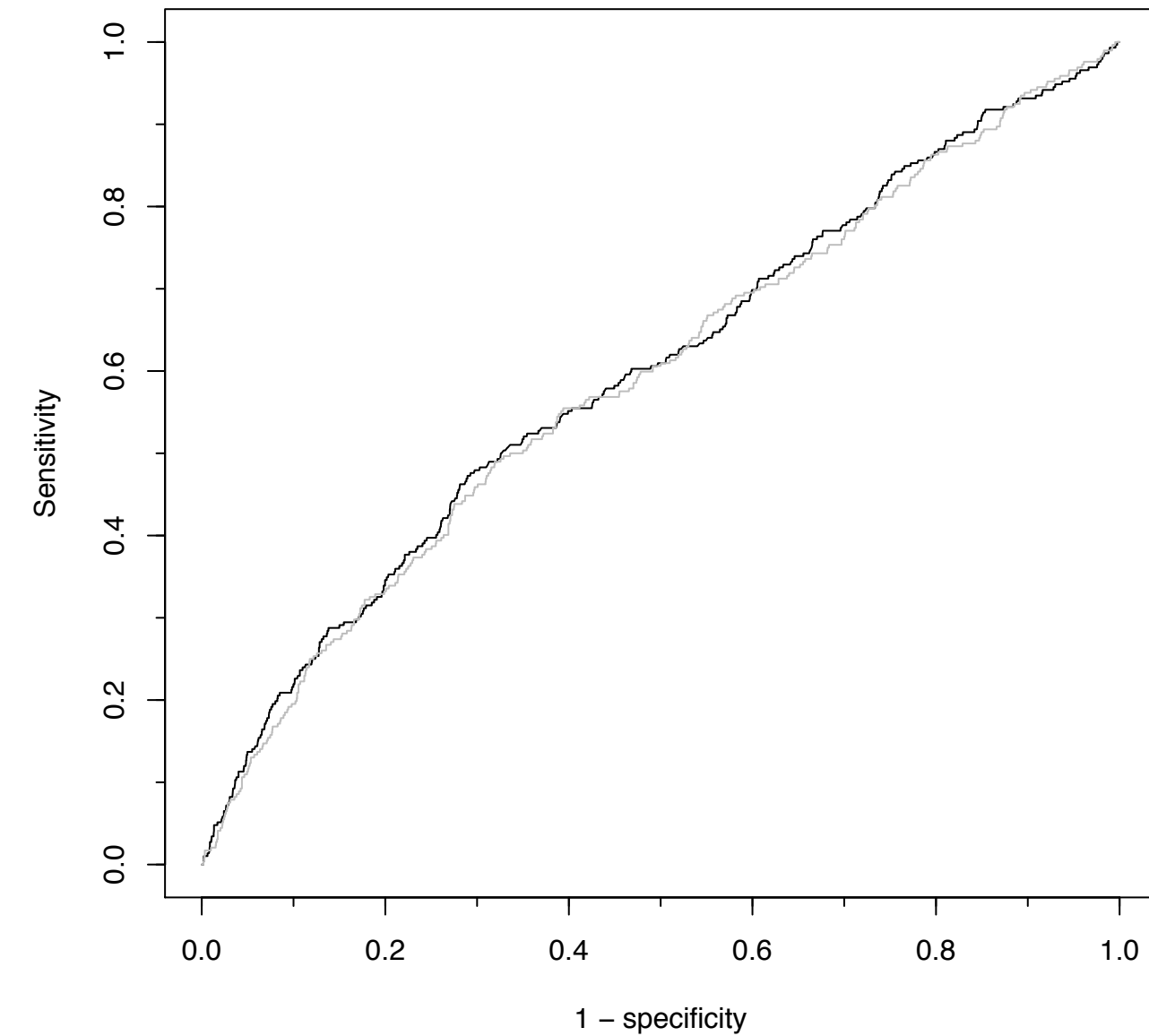

**CT**

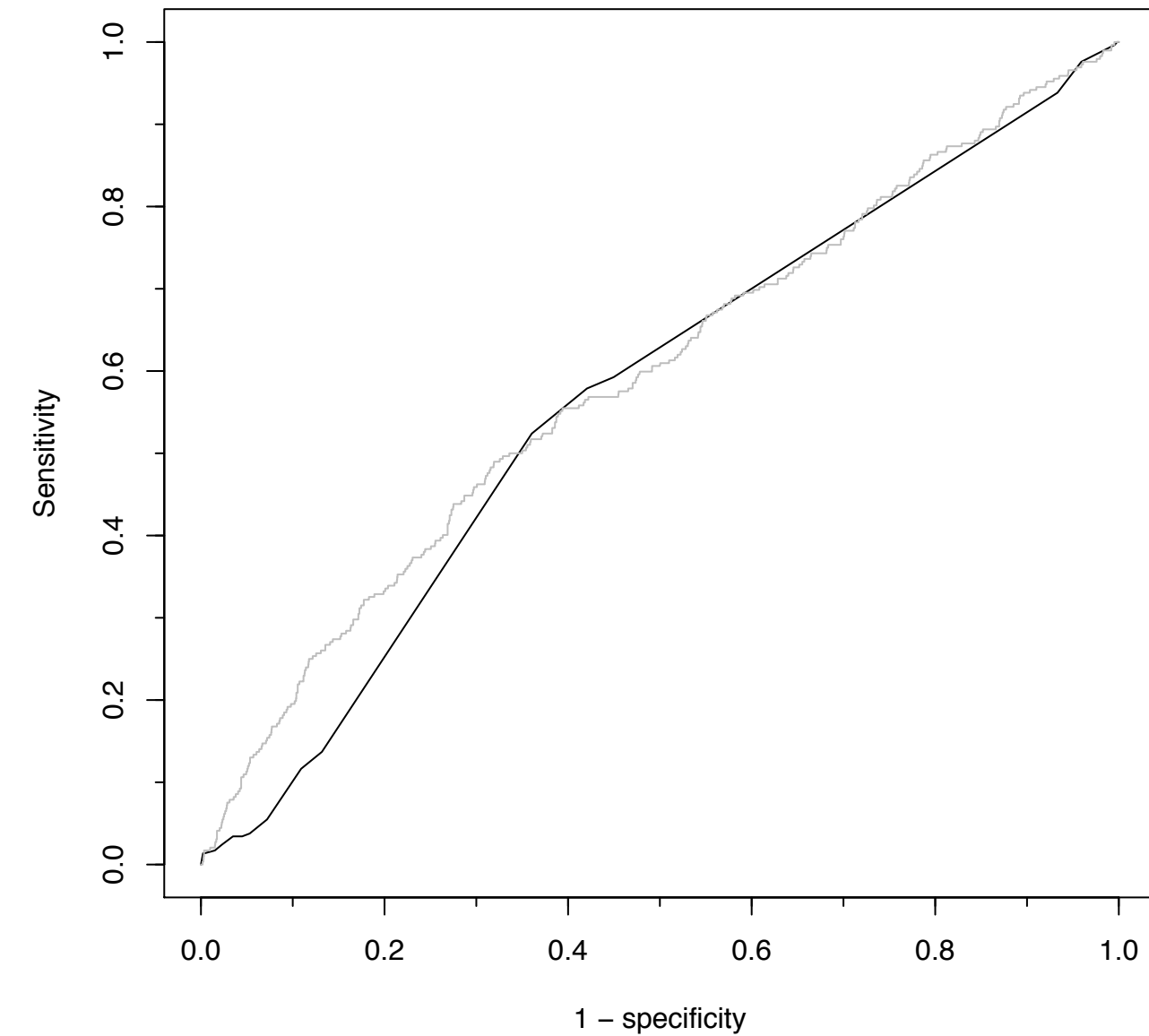

**RF**

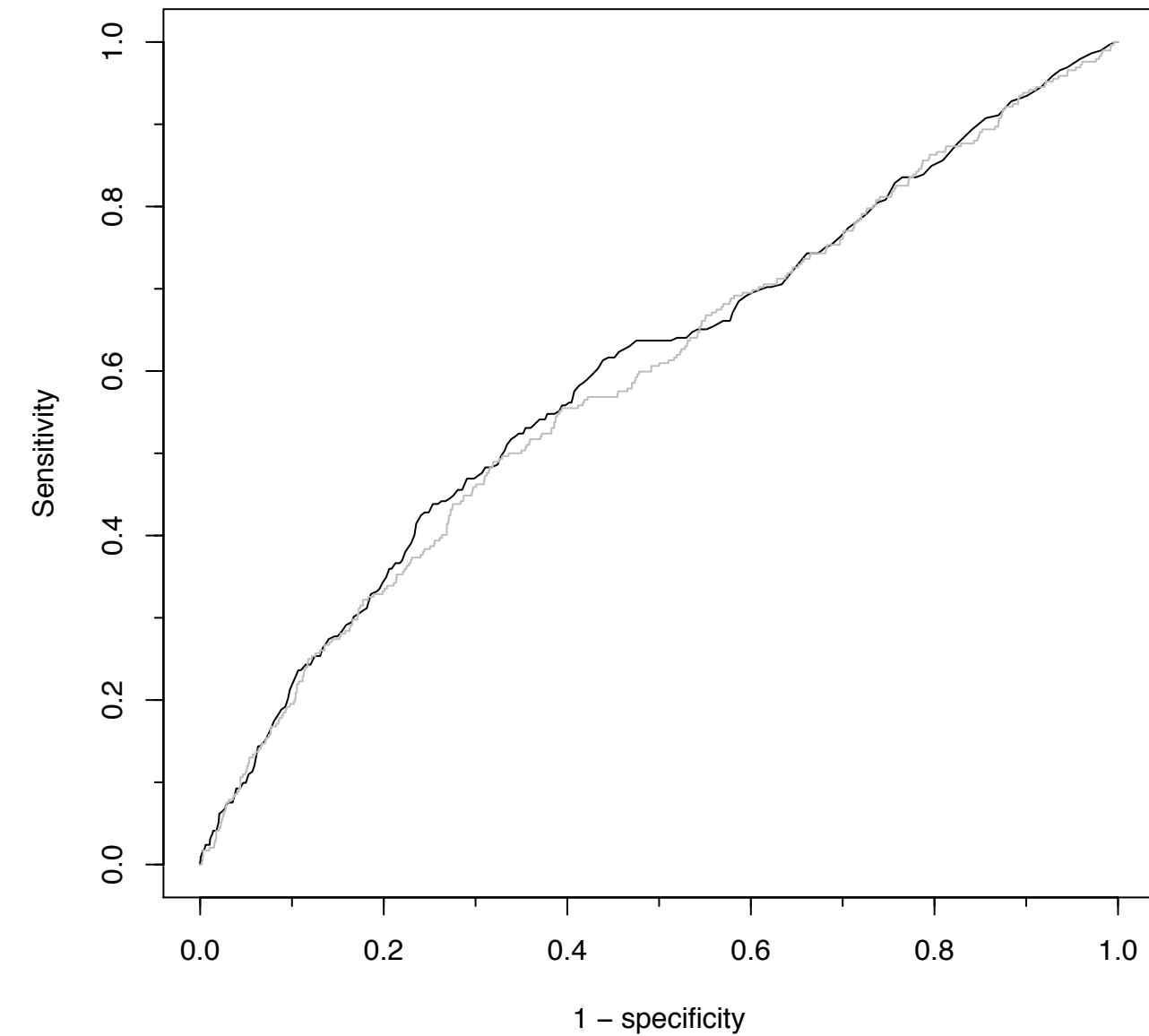

**GB**

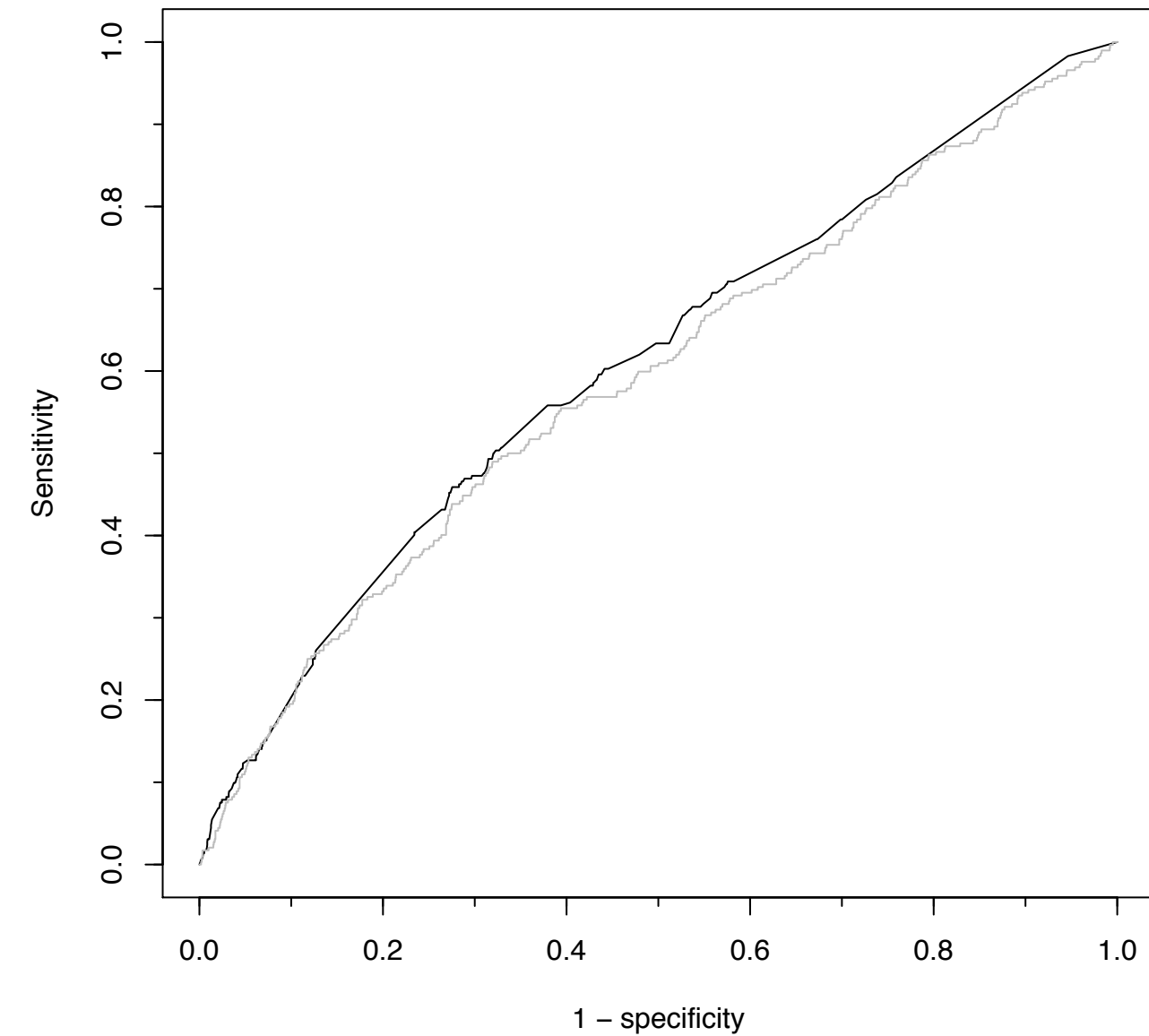

**NN**

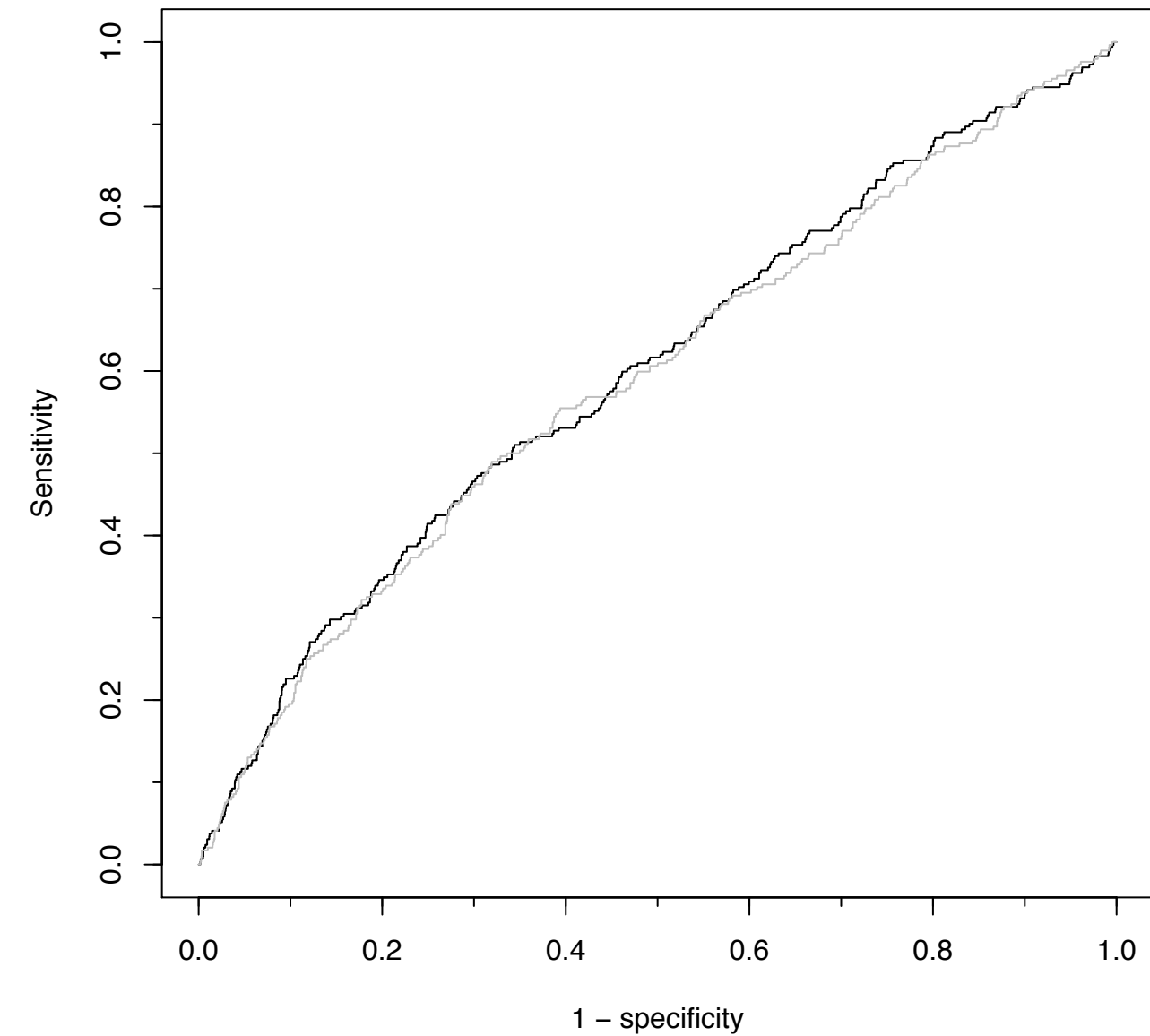

Supplement: Supplementary file 4 — Figure S1. Receiver operating characteristic curves for the prediction of SGA among primiparous women (pre-pregnancy) using elastic net, classification trees, random forest, gradient boosting, and neural networks. (PDF 176 kb) [file 12884_2018_1971_MOESM4_ESM.pdf]

**EN**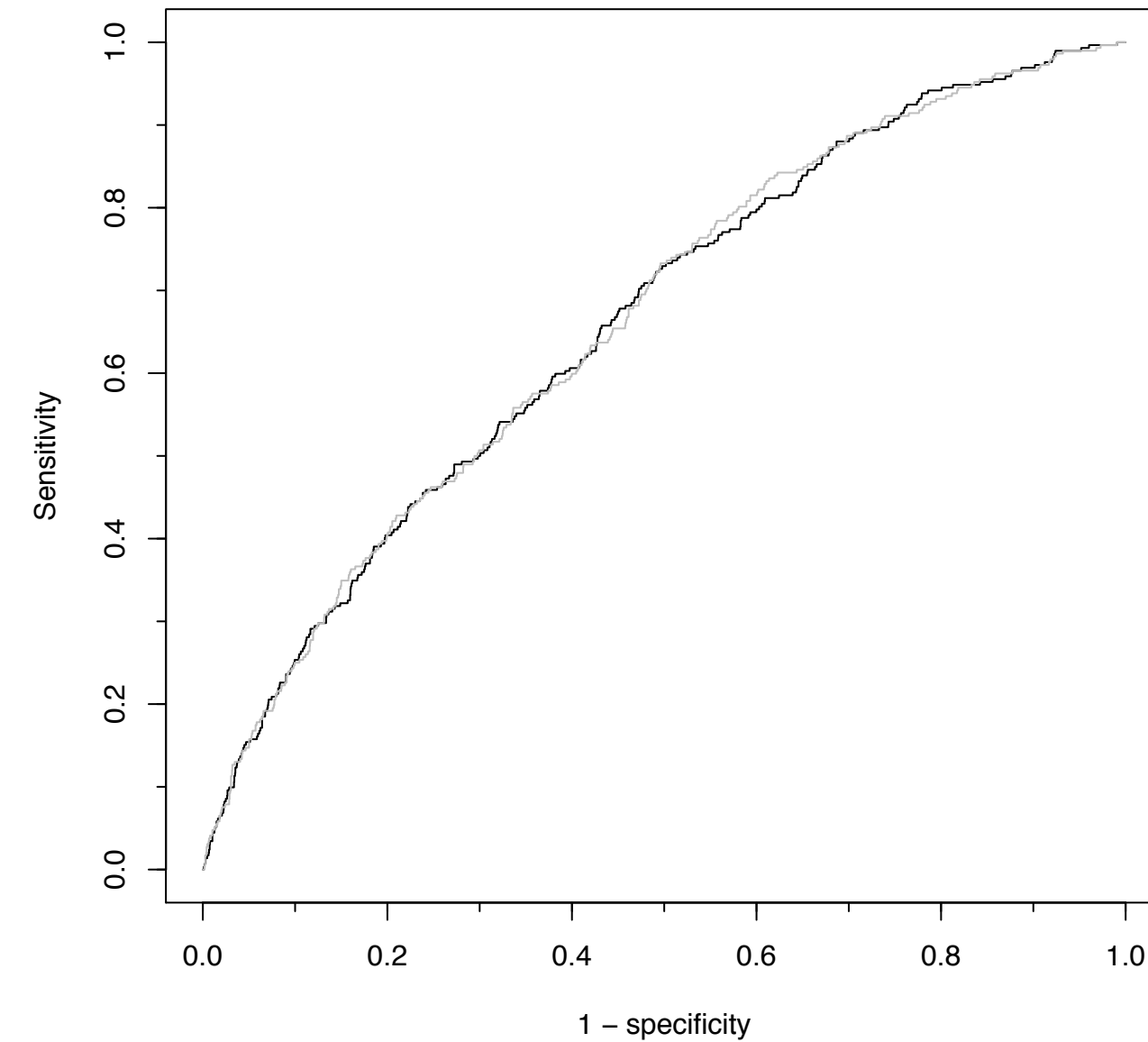**CT**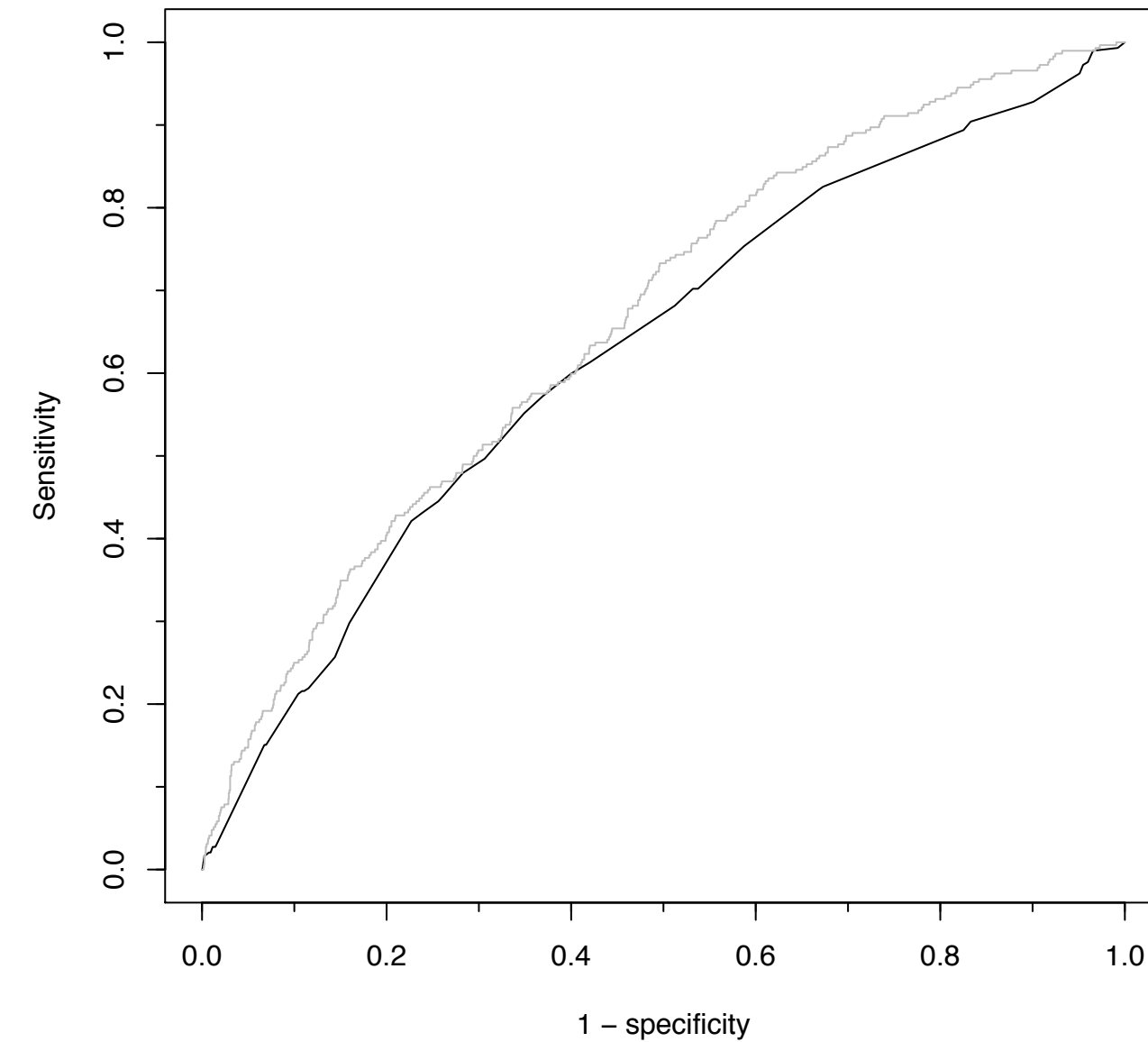**RF**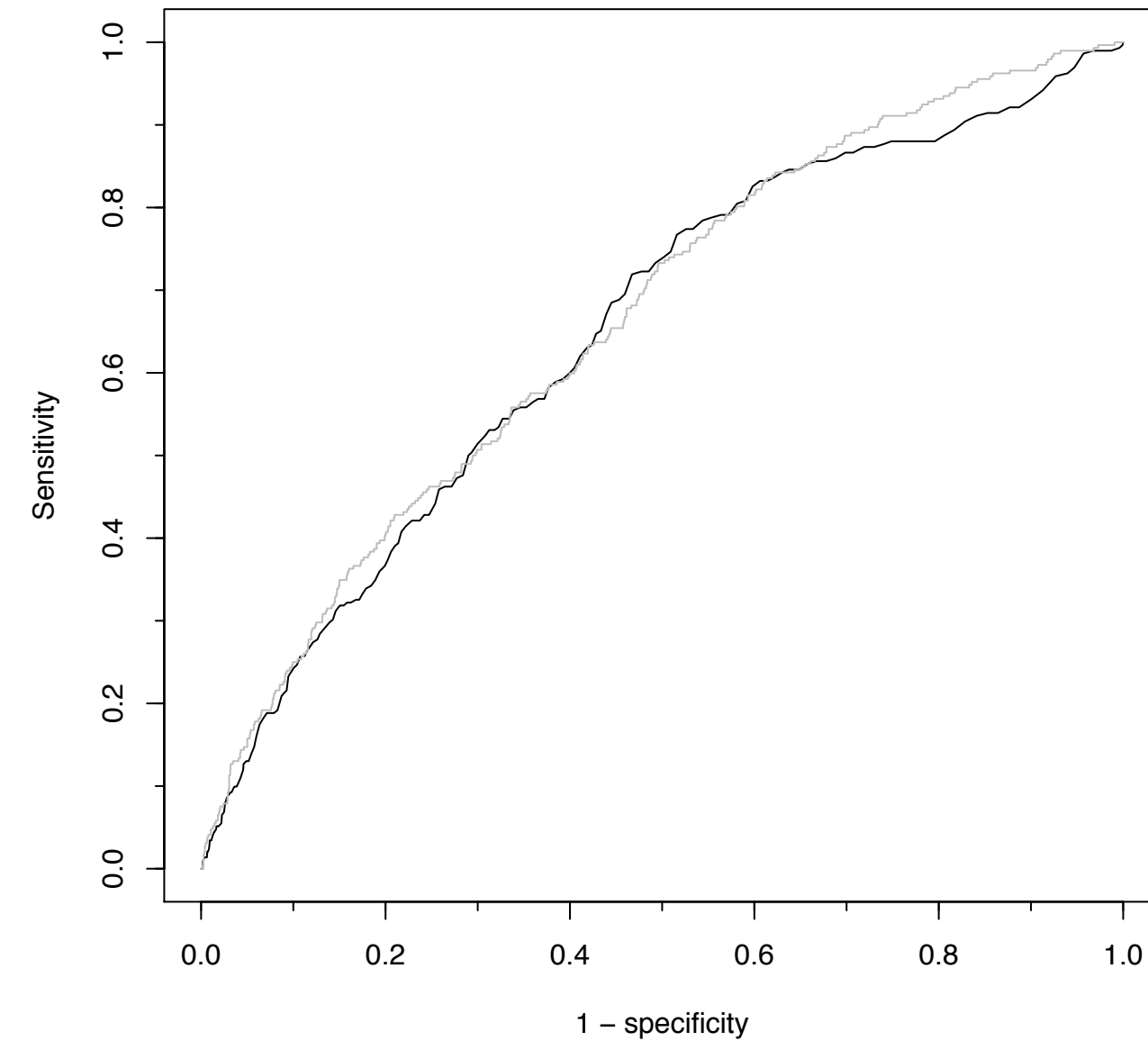**GB**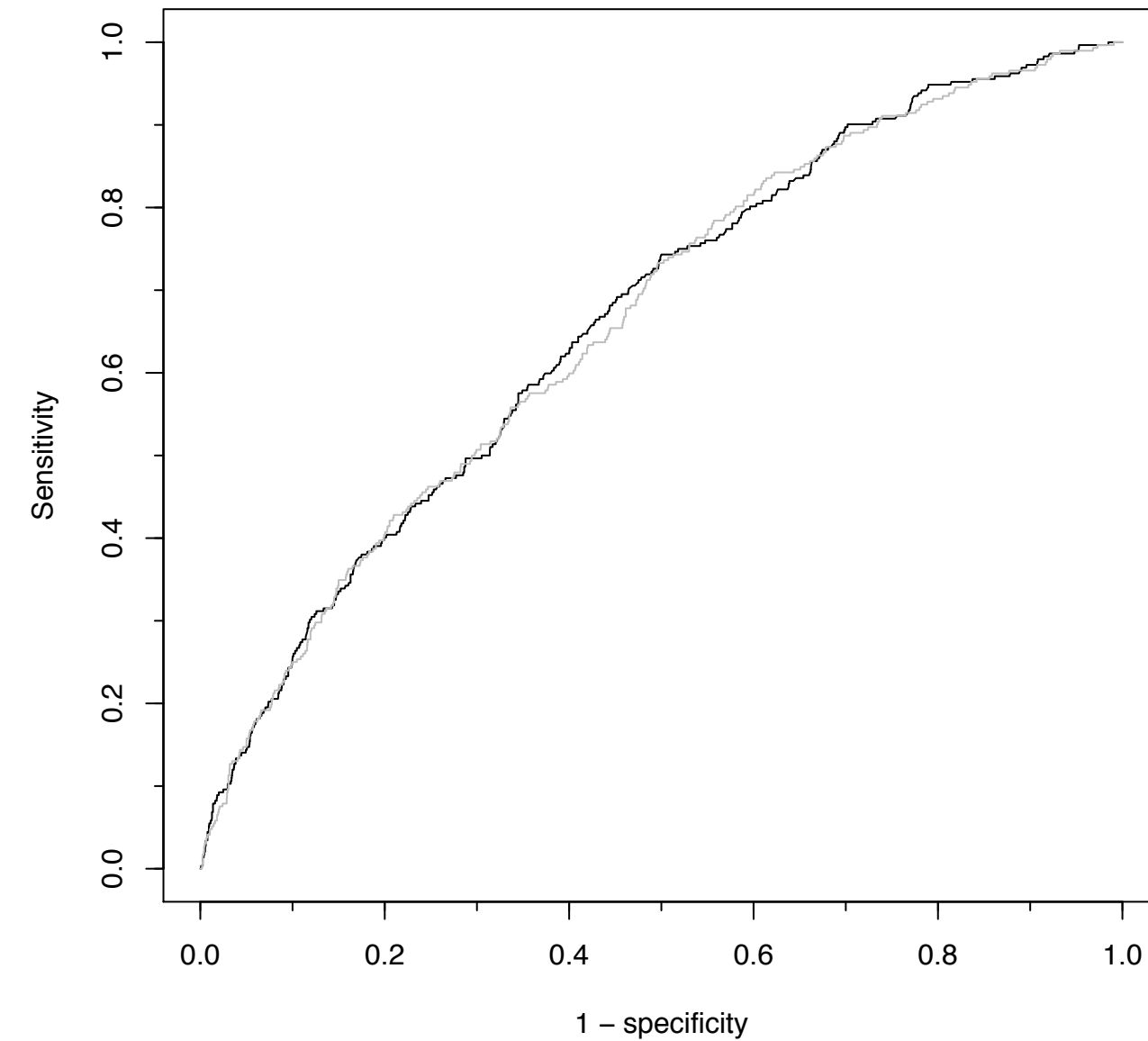**NN**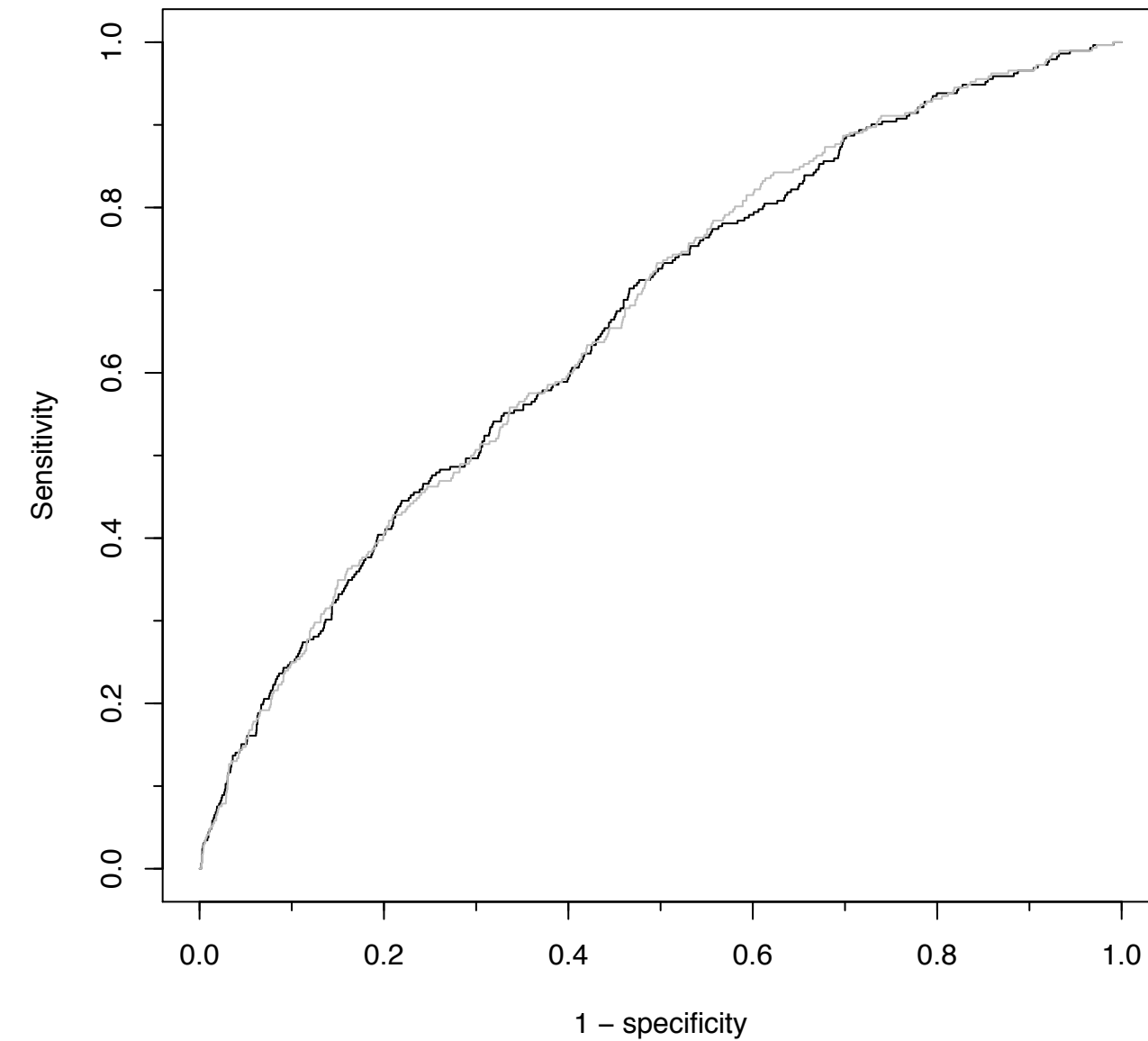

Supplement: Supplementary file 5 — Figure S2. Receiver operating characteristic curves for the prediction of SGA among primiparous women (26 weeks) using elastic net, classification trees, random forest, gradient boosting, and neural networks. (PDF 186 kb) [file 12884_2018_1971_MOESM5_ESM.pdf]

**EN**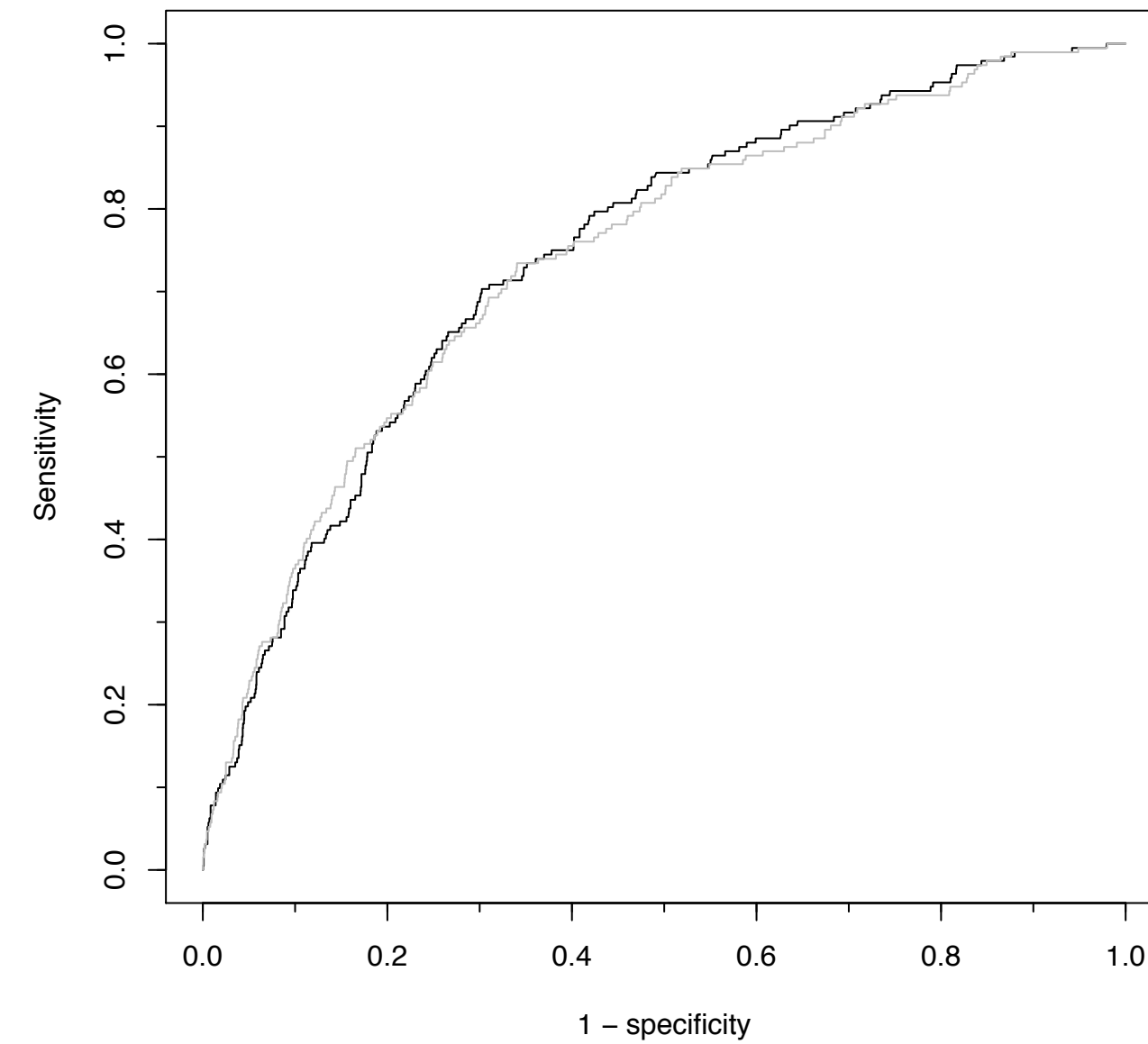**CT**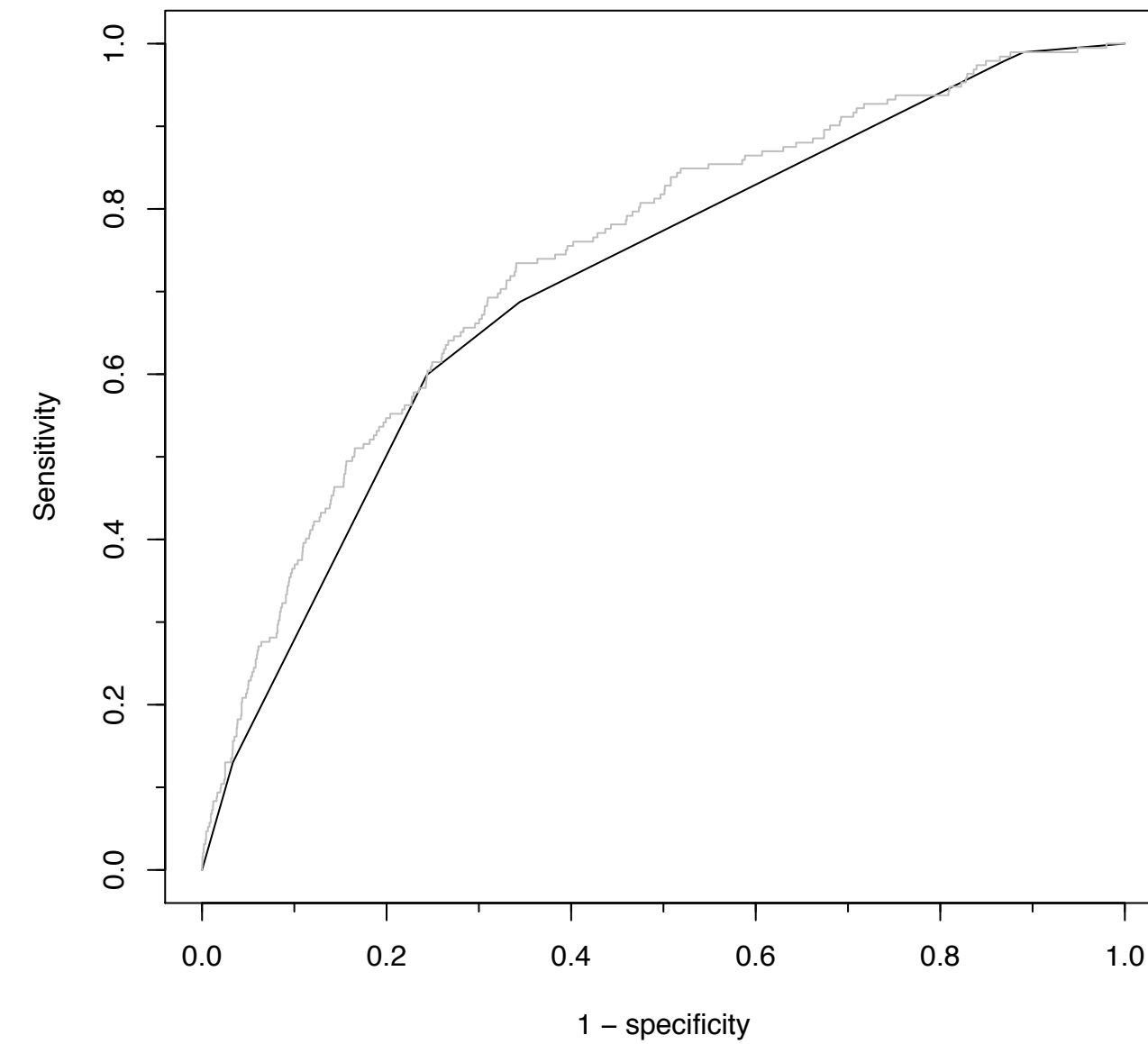**RF**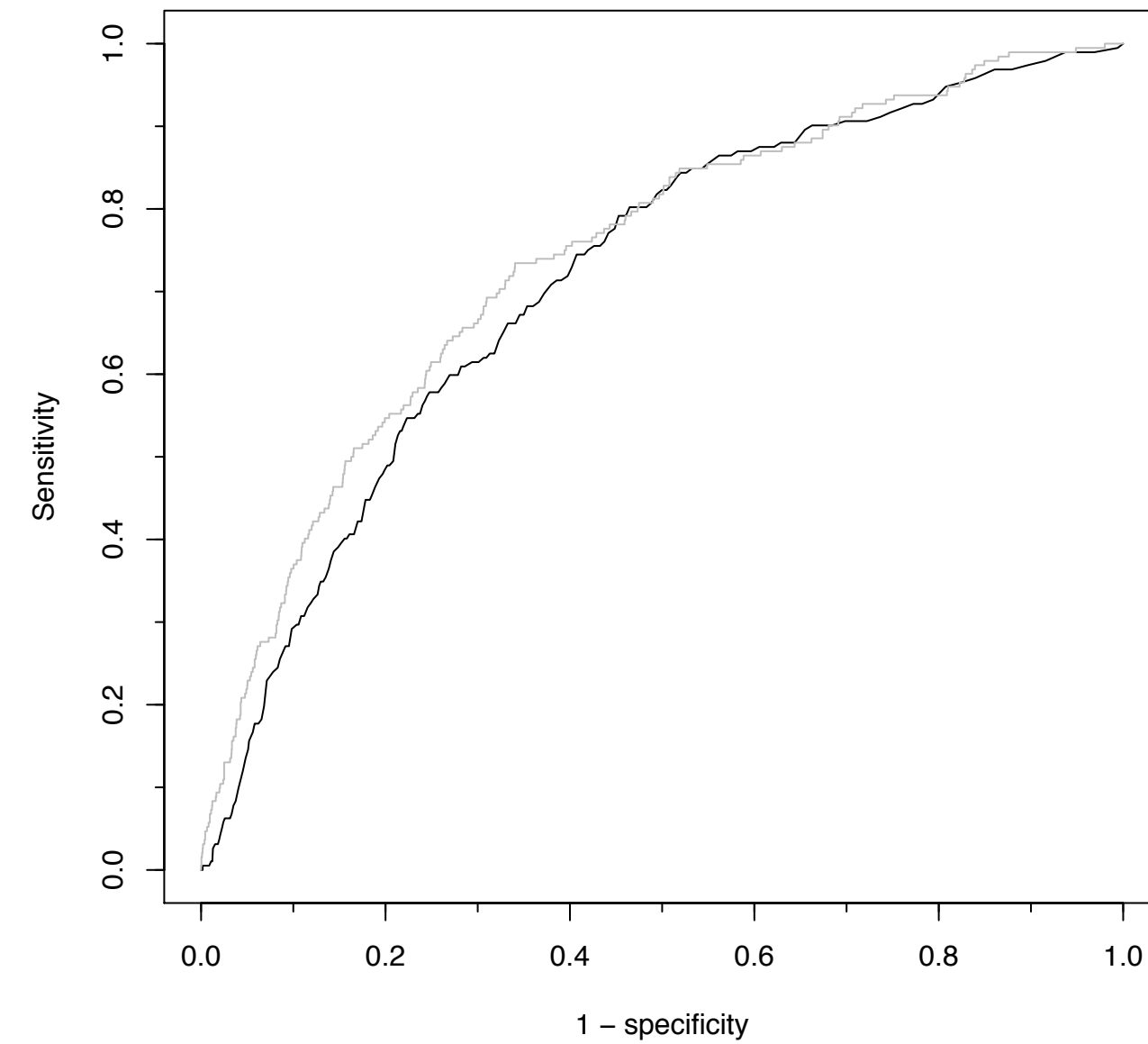**GB**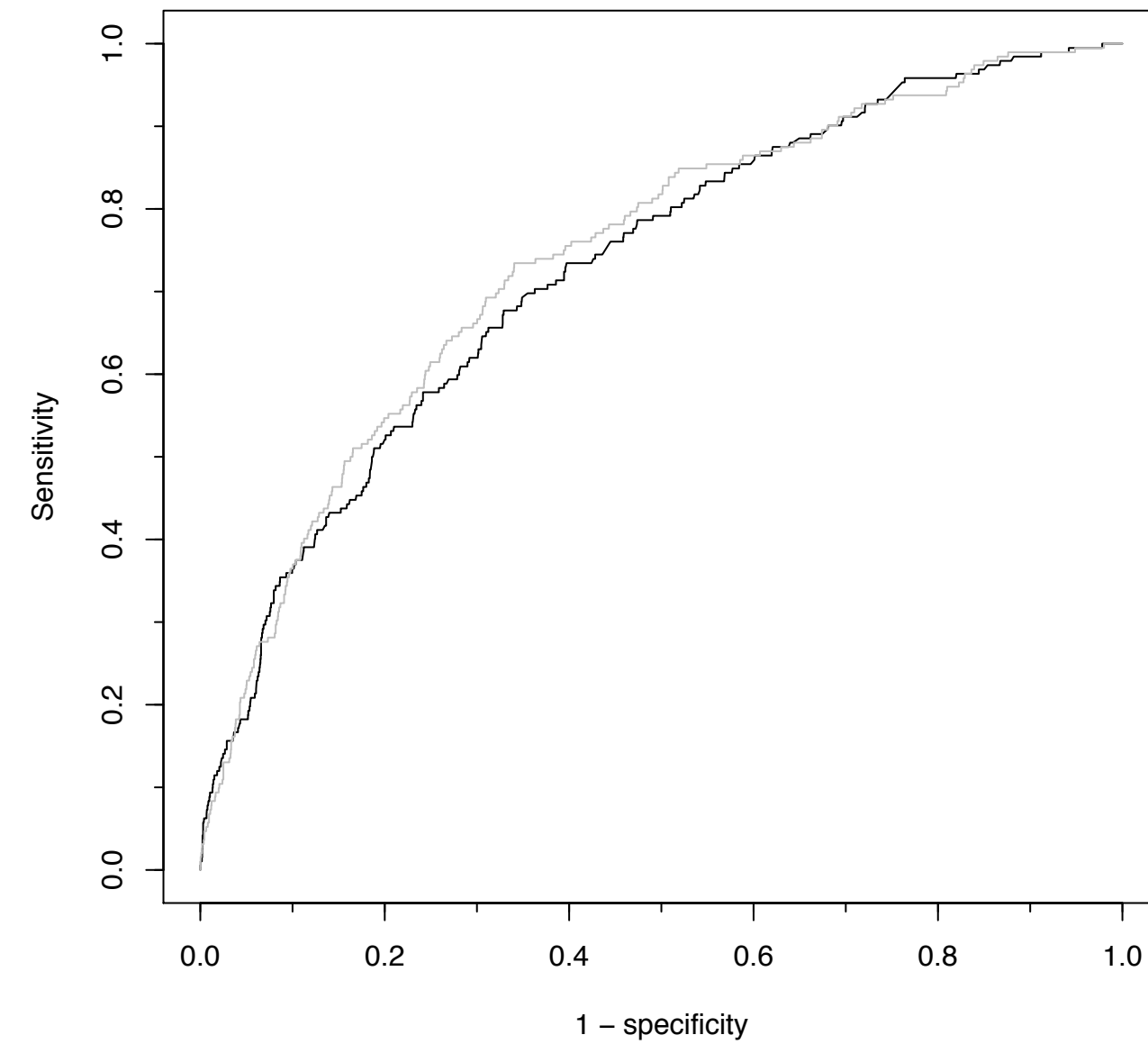**NN**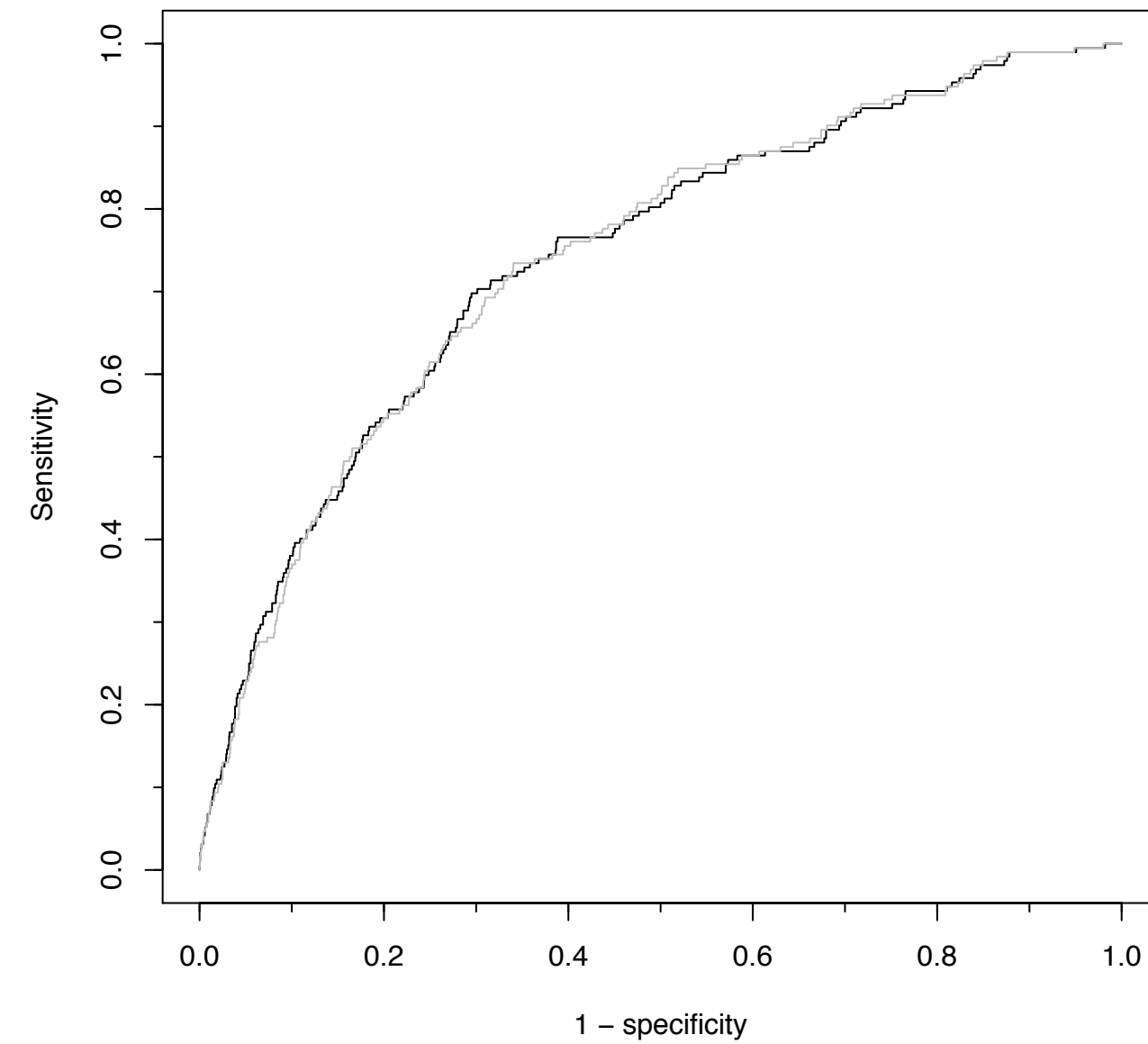

Supplement: Supplementary file 6 — Figure S3. Receiver operating characteristic curves for the prediction of SGA among multiparous women (pre-pregnancy) using elastic net, classification trees, random forest, gradient boosting, and neural networks. (PDF 188 kb) [file 12884_2018_1971_MOESM6_ESM.pdf]

**EN**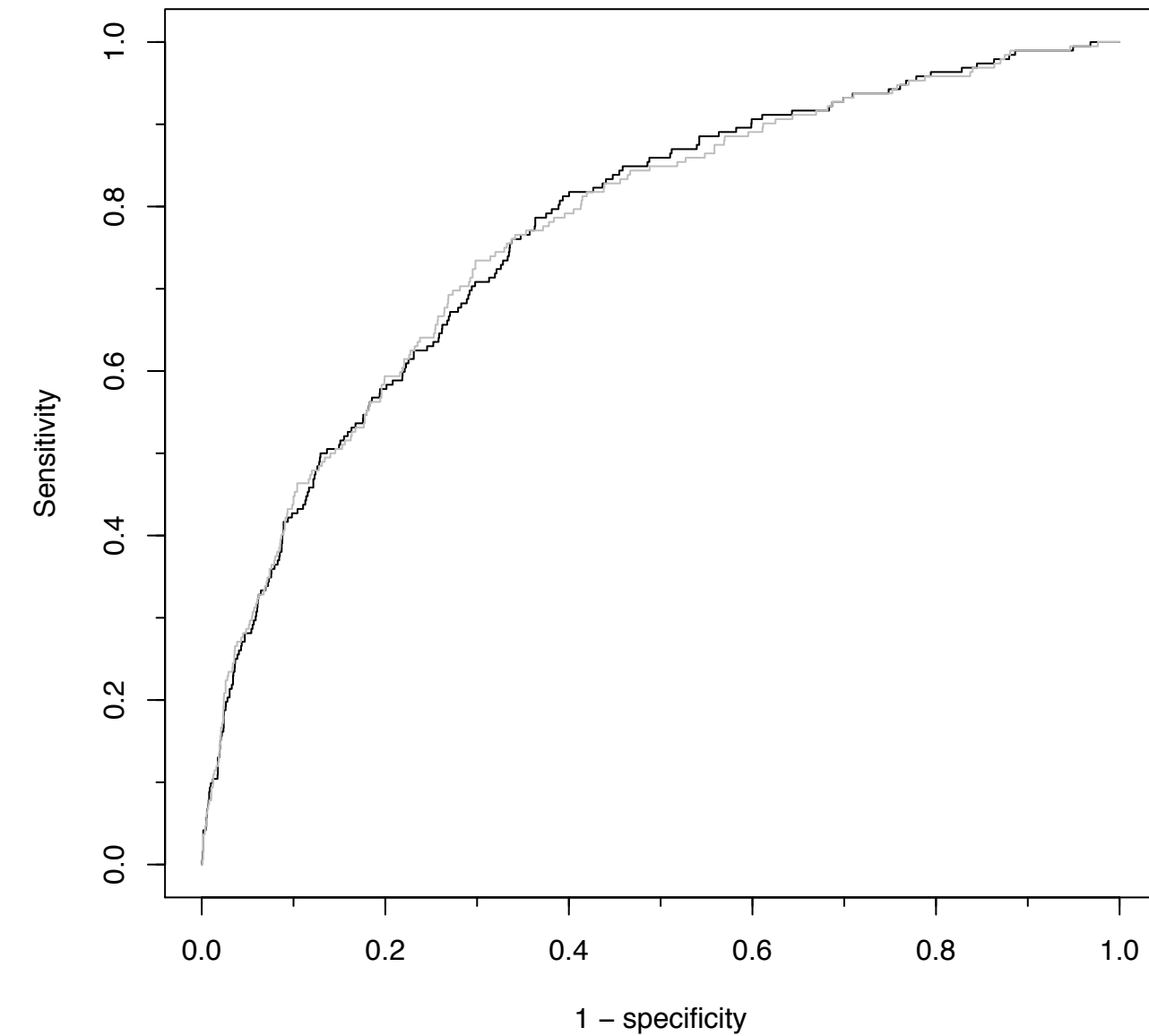**CT**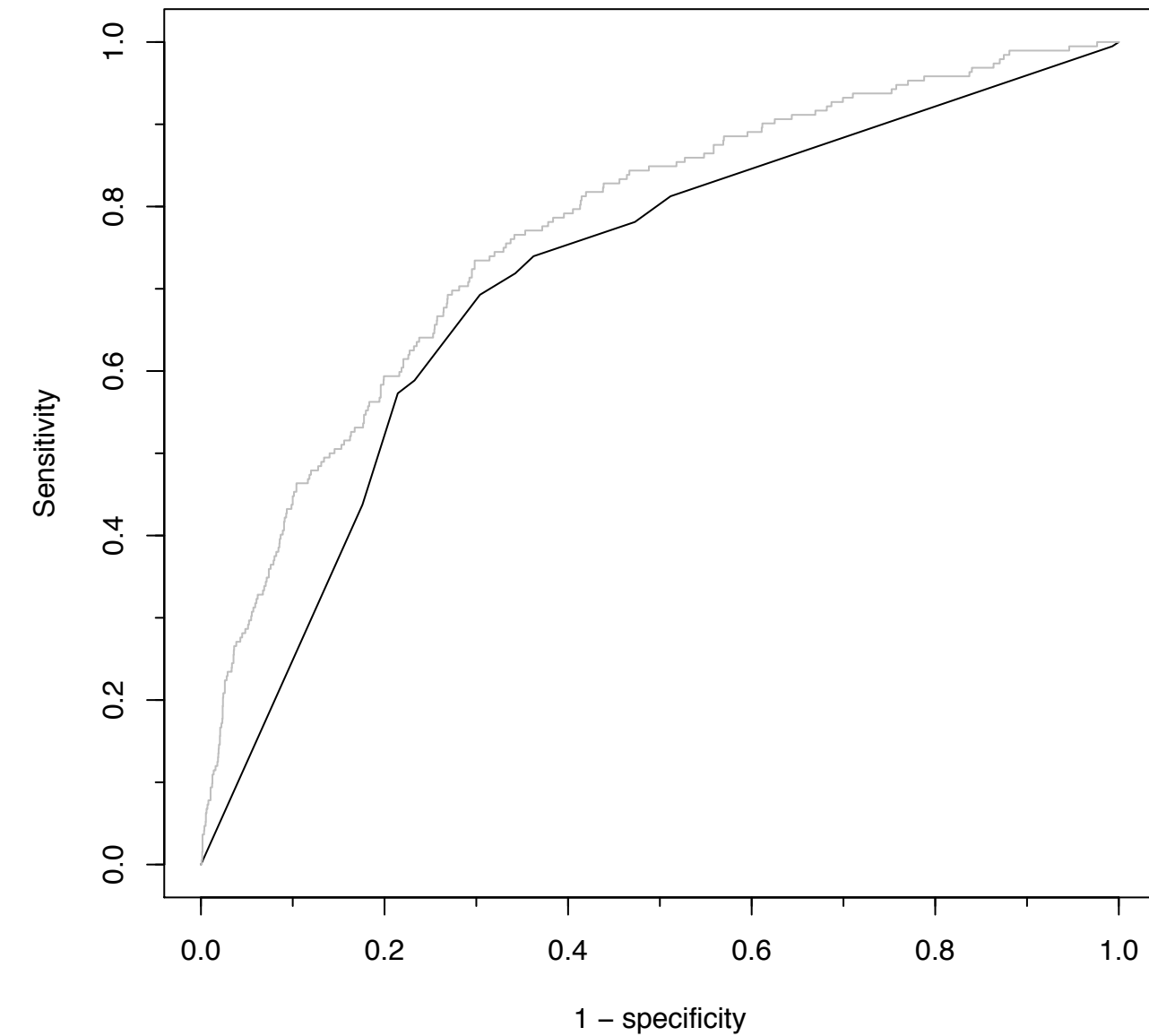**RF**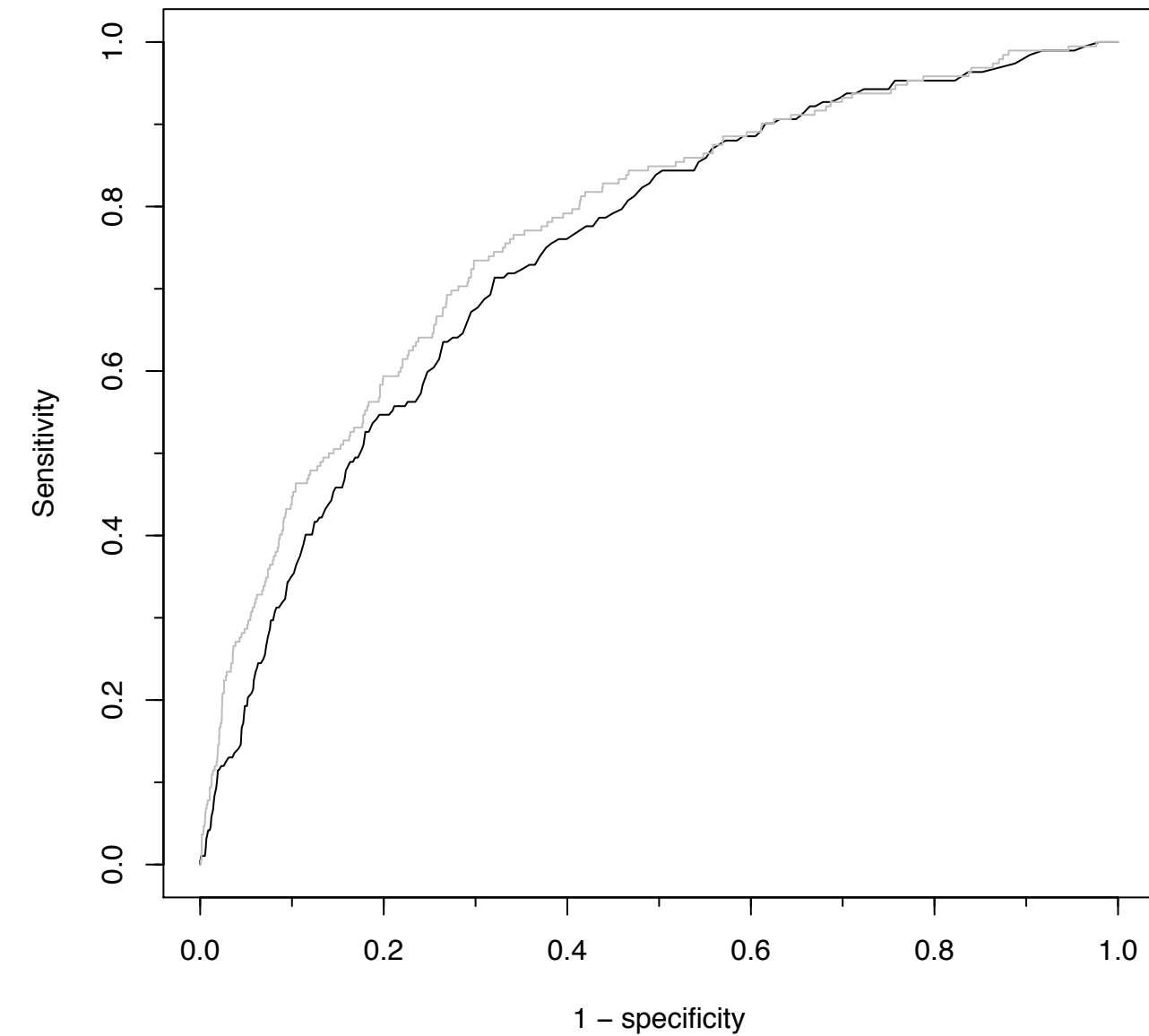**GB**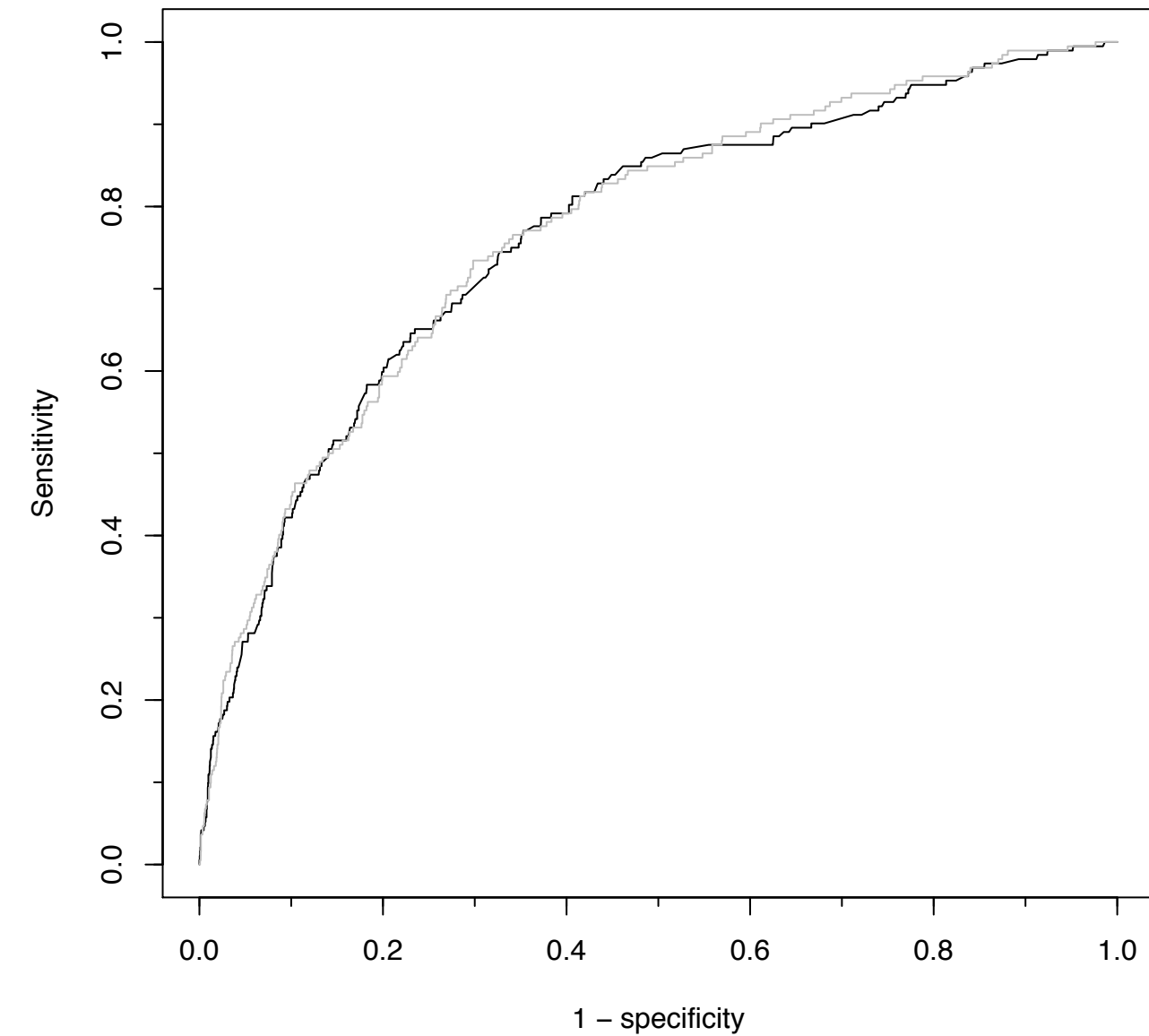**NN**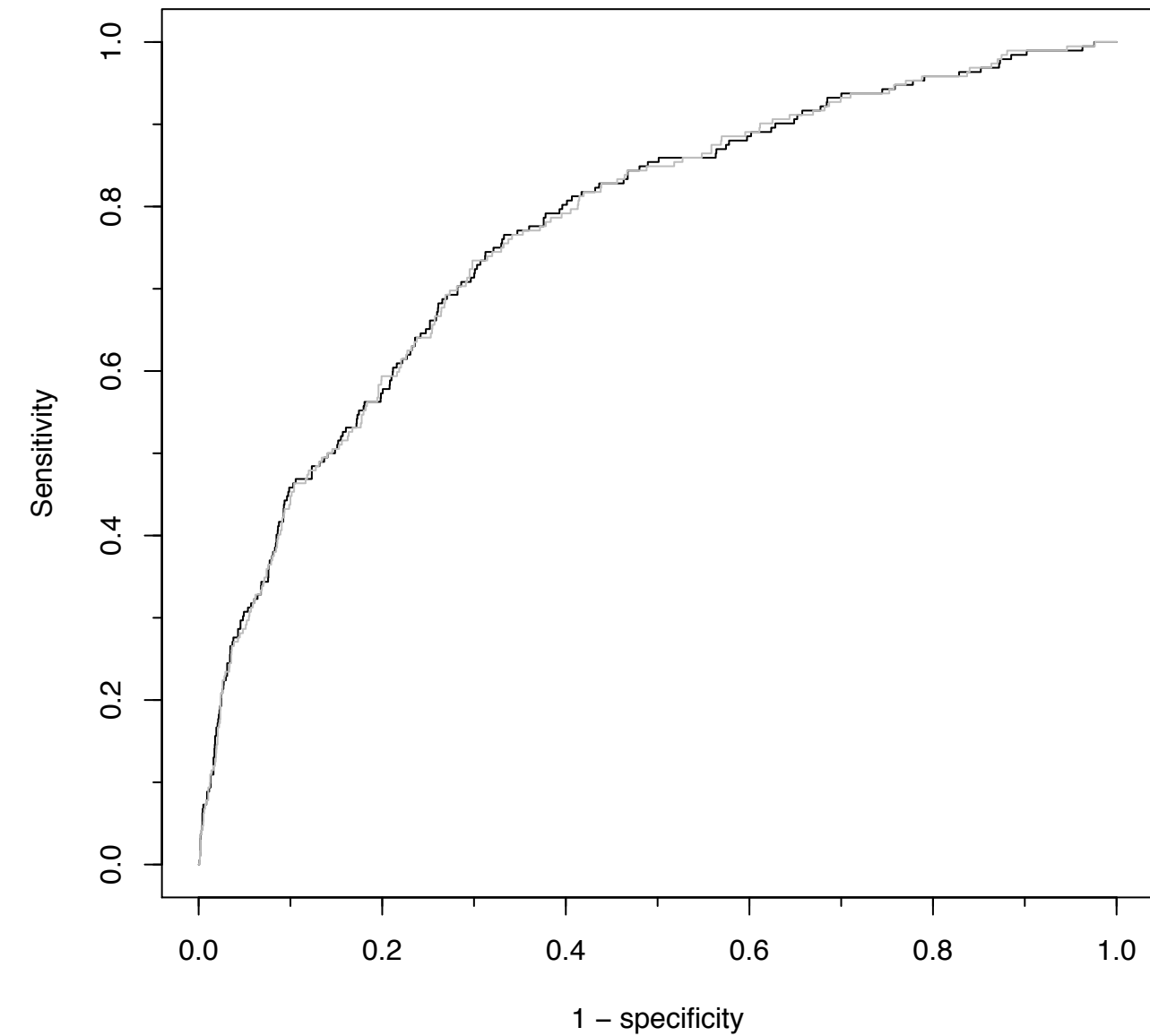

Supplement: Supplementary file 7 — Figure S4. Receiver operating characteristic curves for the prediction of SGA among multiparous women (26 weeks) using elastic net, classification trees, random forest, gradient boosting, and neural networks. (PDF 189 kb) [file 12884_2018_1971_MOESM7_ESM.pdf]

**EN**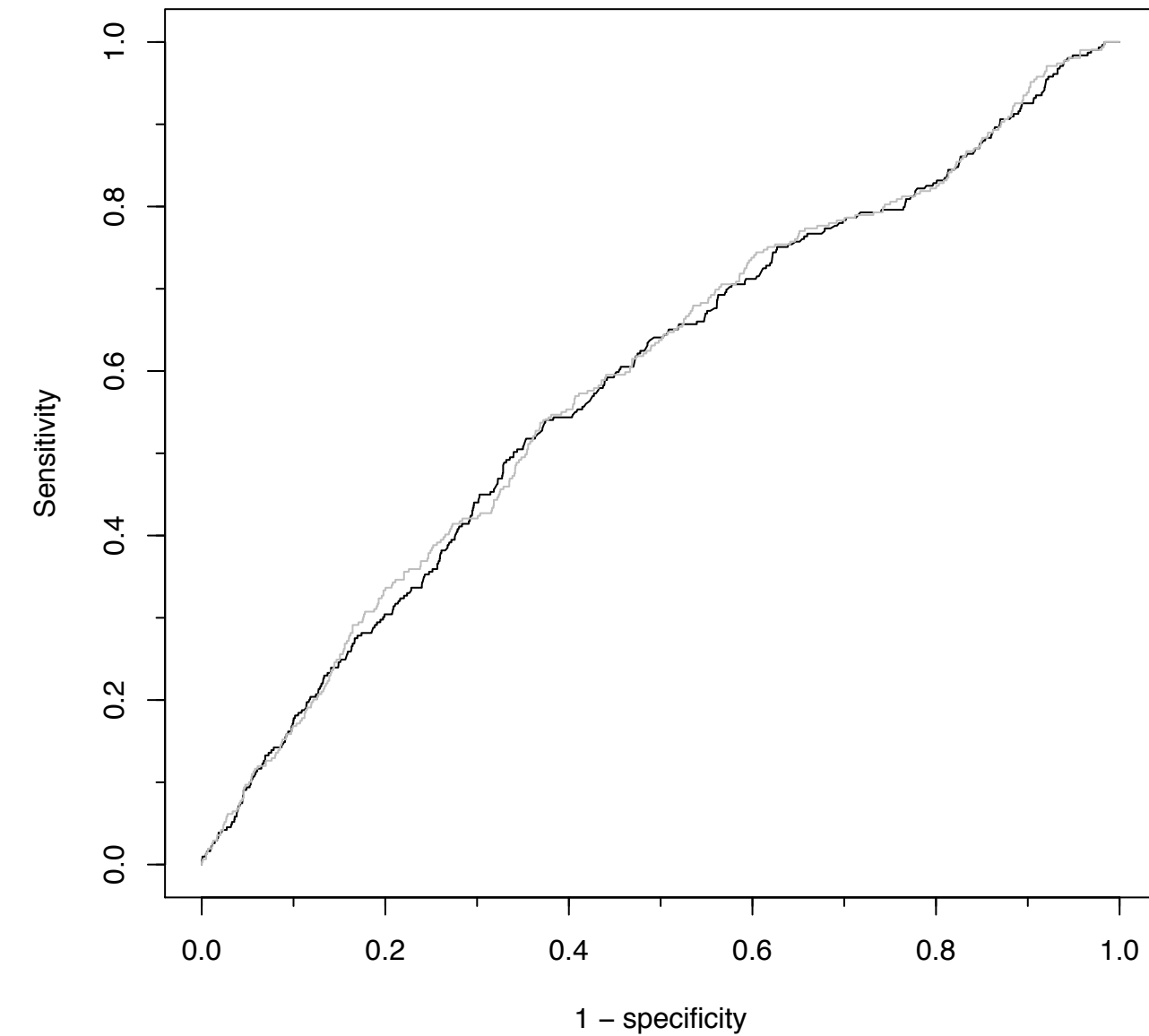**CT**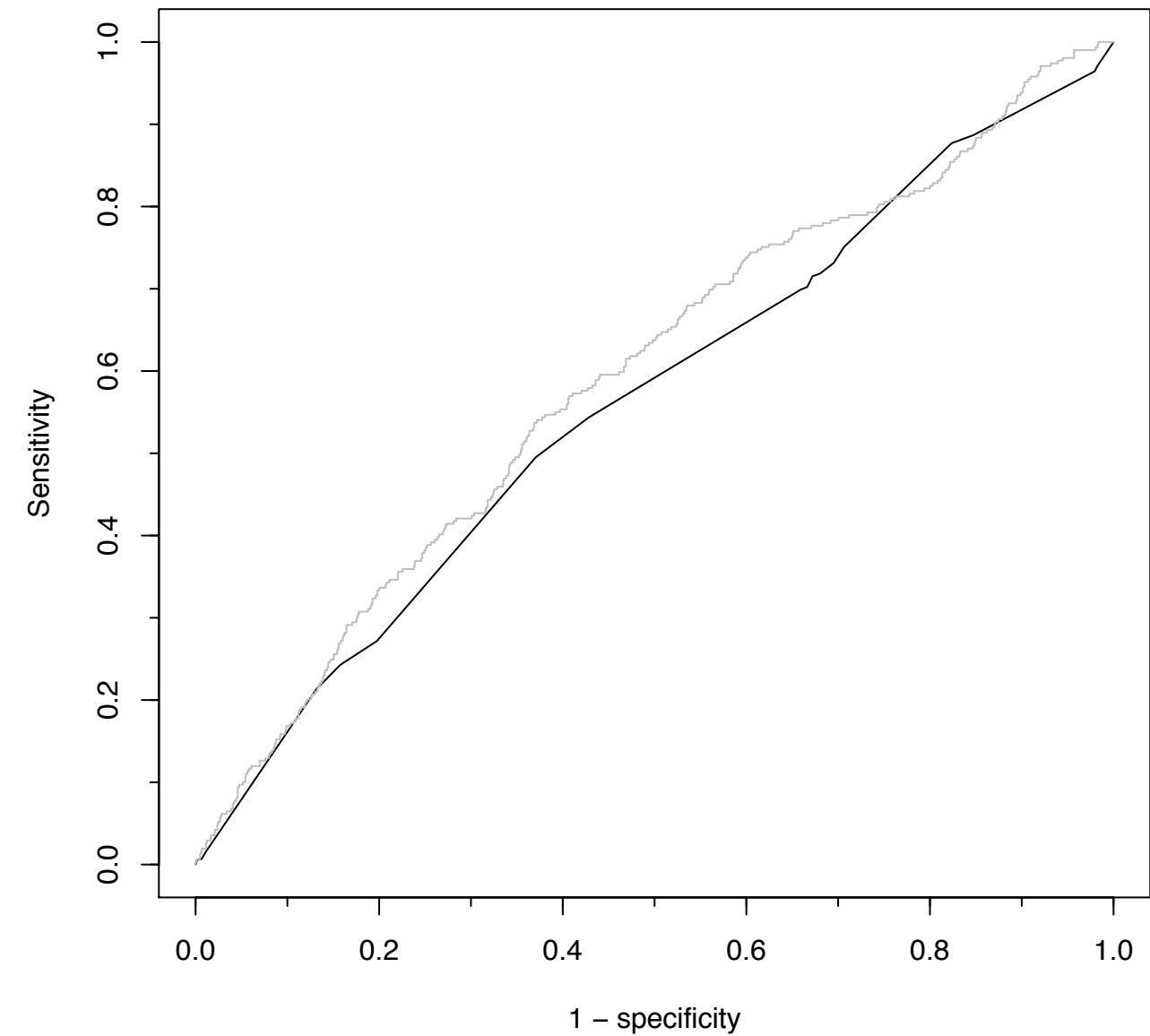**RF**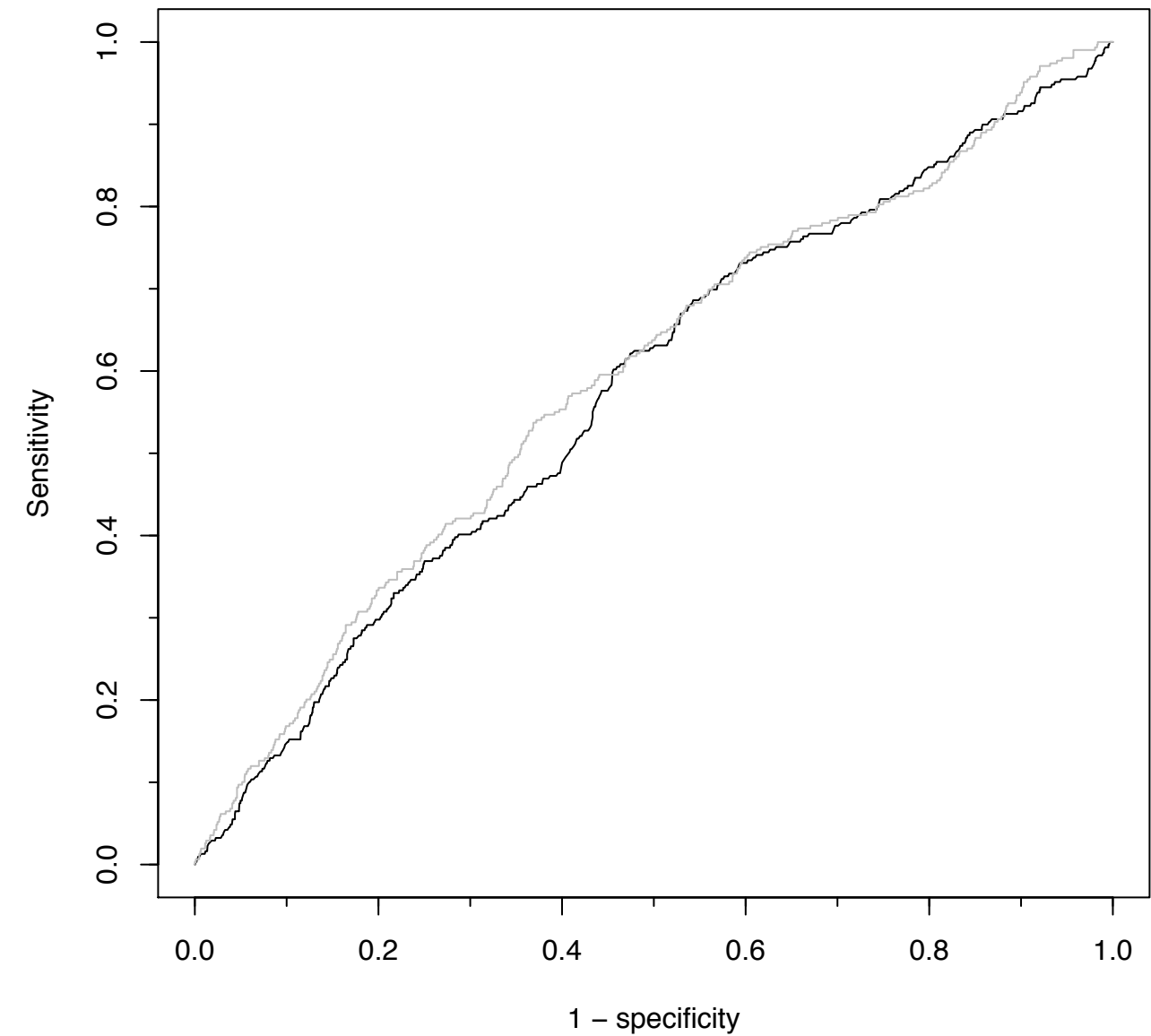**GB**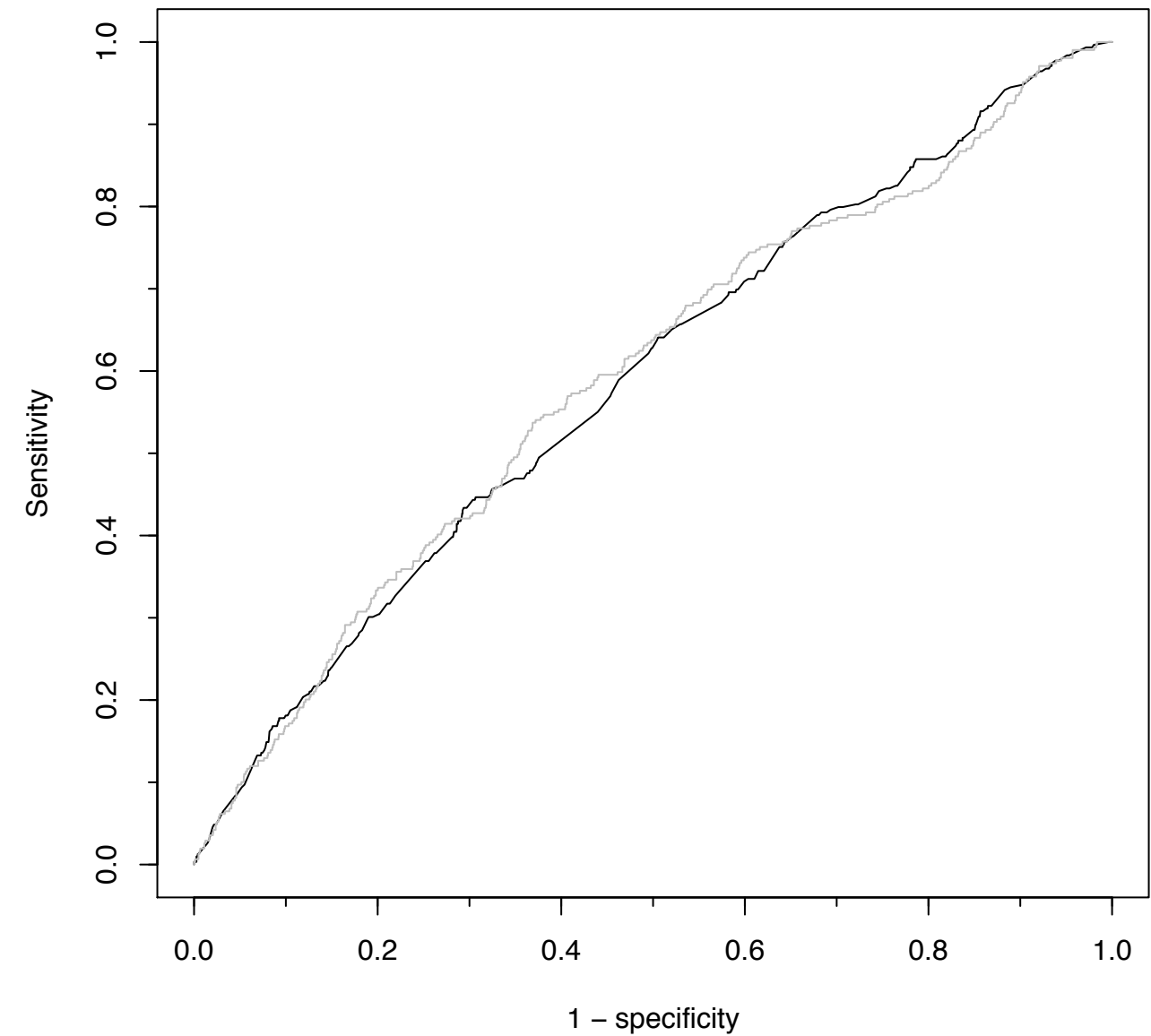**NN**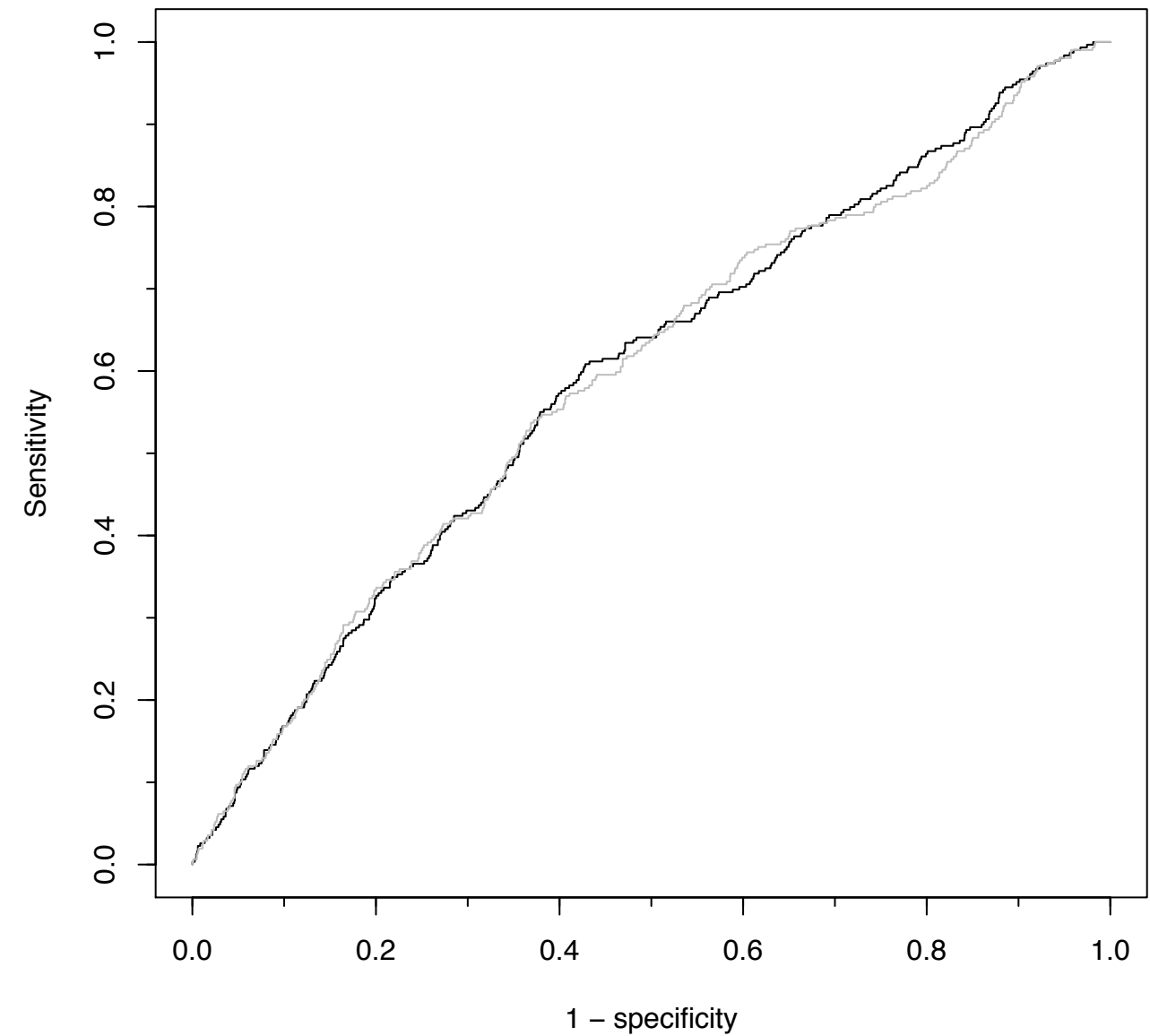

Supplement: Supplementary file 8 — Figure S5. Receiver operating characteristic curves for the prediction of LGA among primiparous women (pre-pregnancy) using elastic net, classification trees, random forest, gradient boosting, and neural networks. (PDF 180 kb) [file 12884_2018_1971_MOESM8_ESM.pdf]

**EN**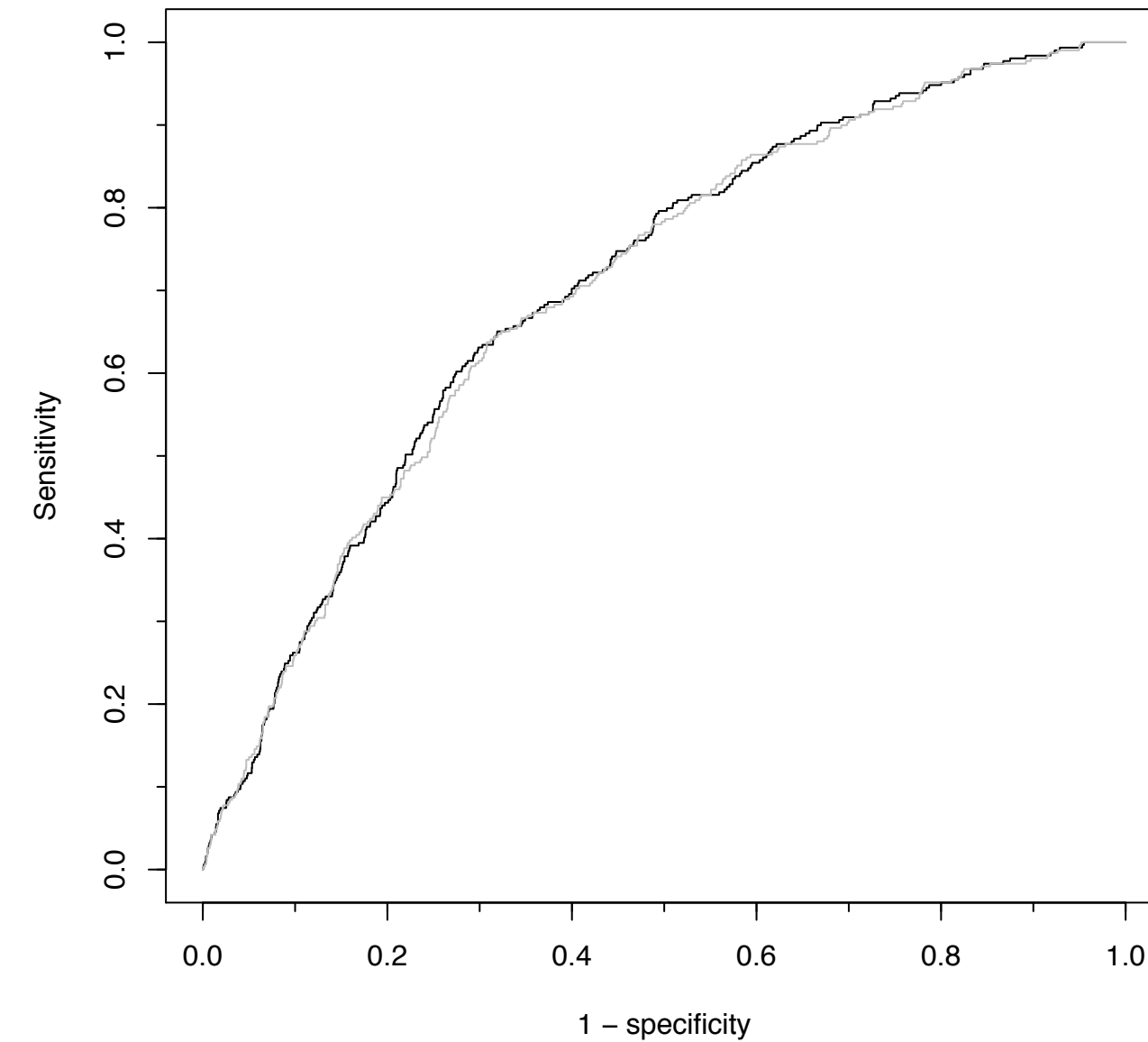**CT**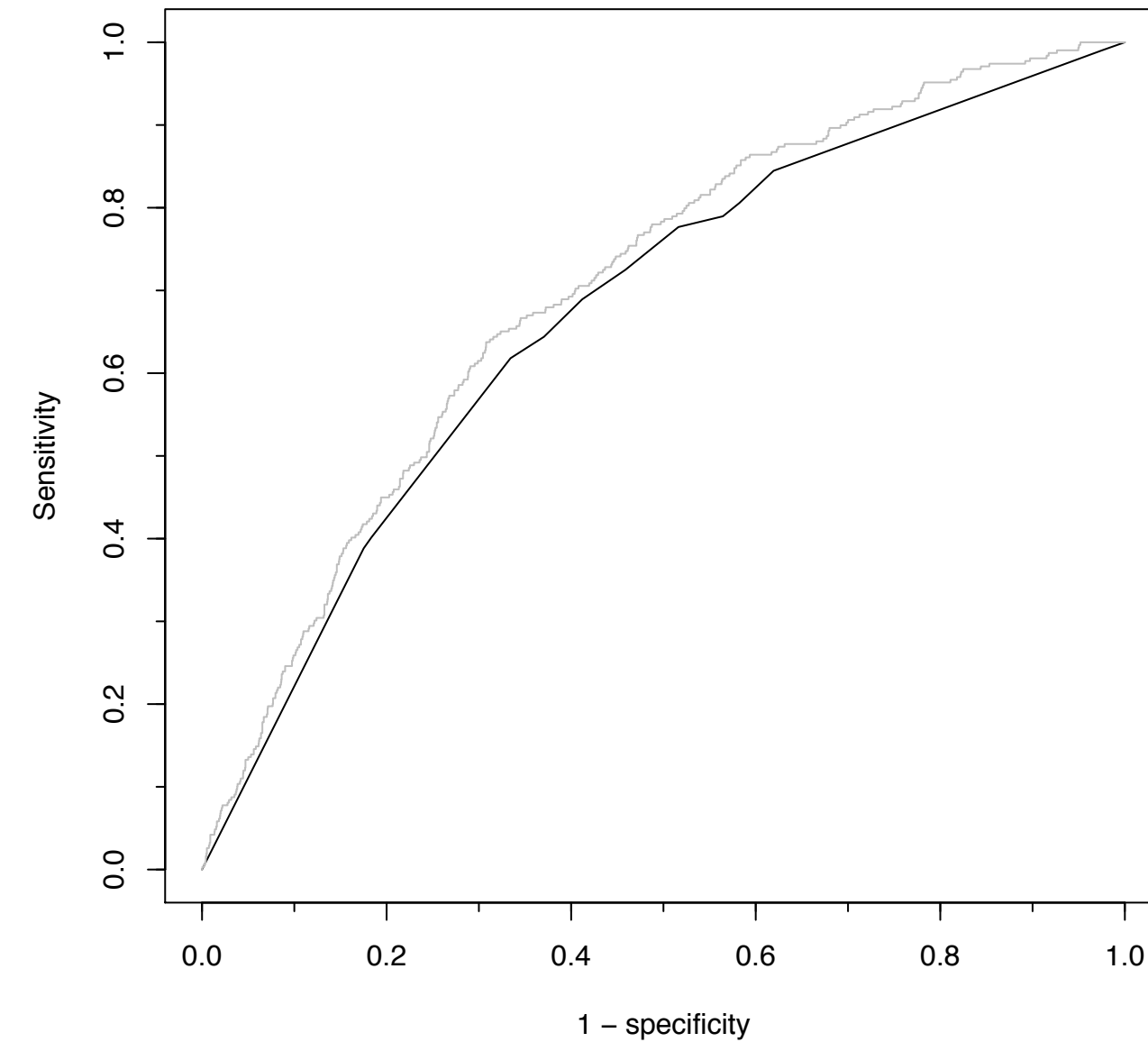**RF**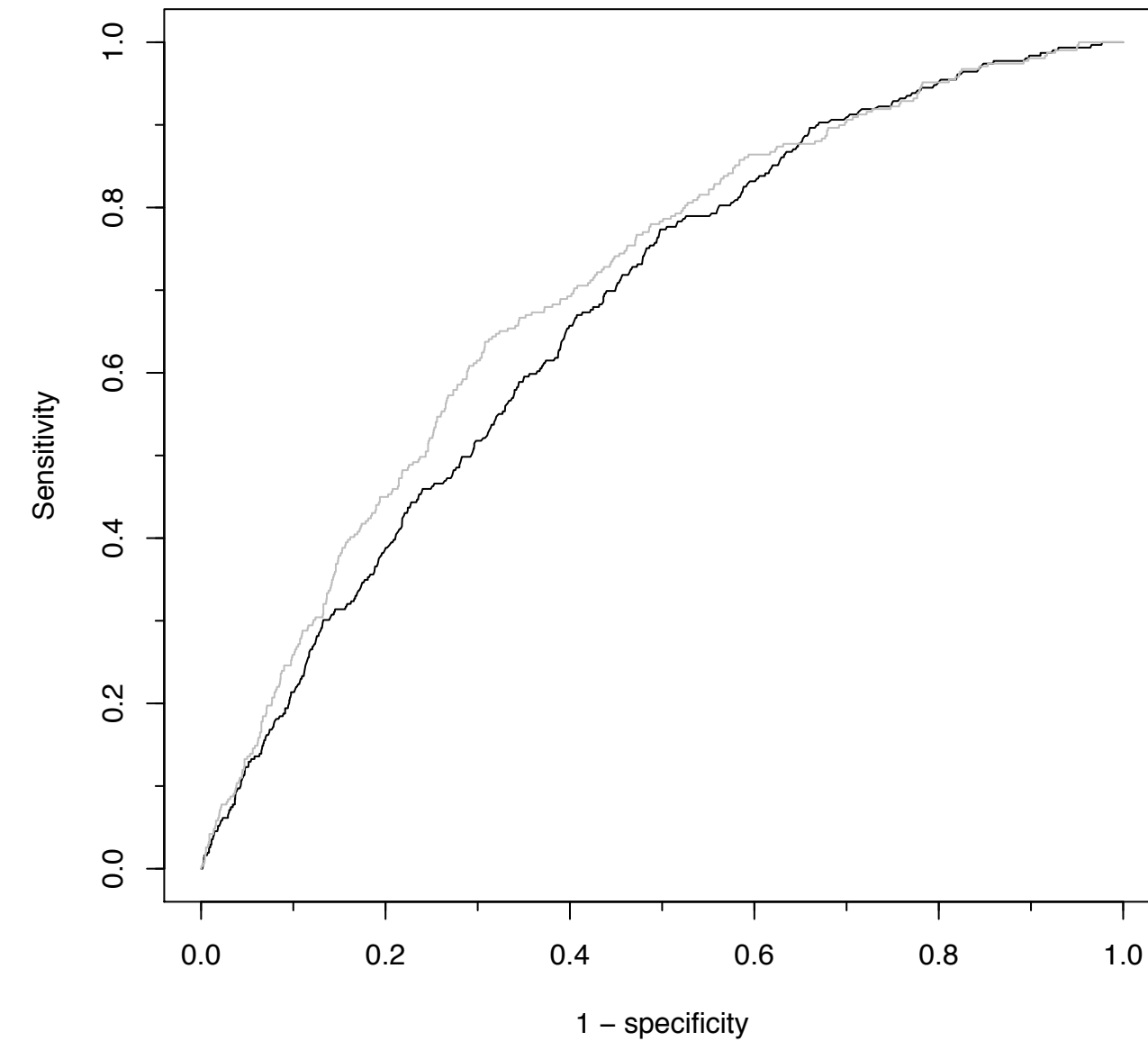**GB**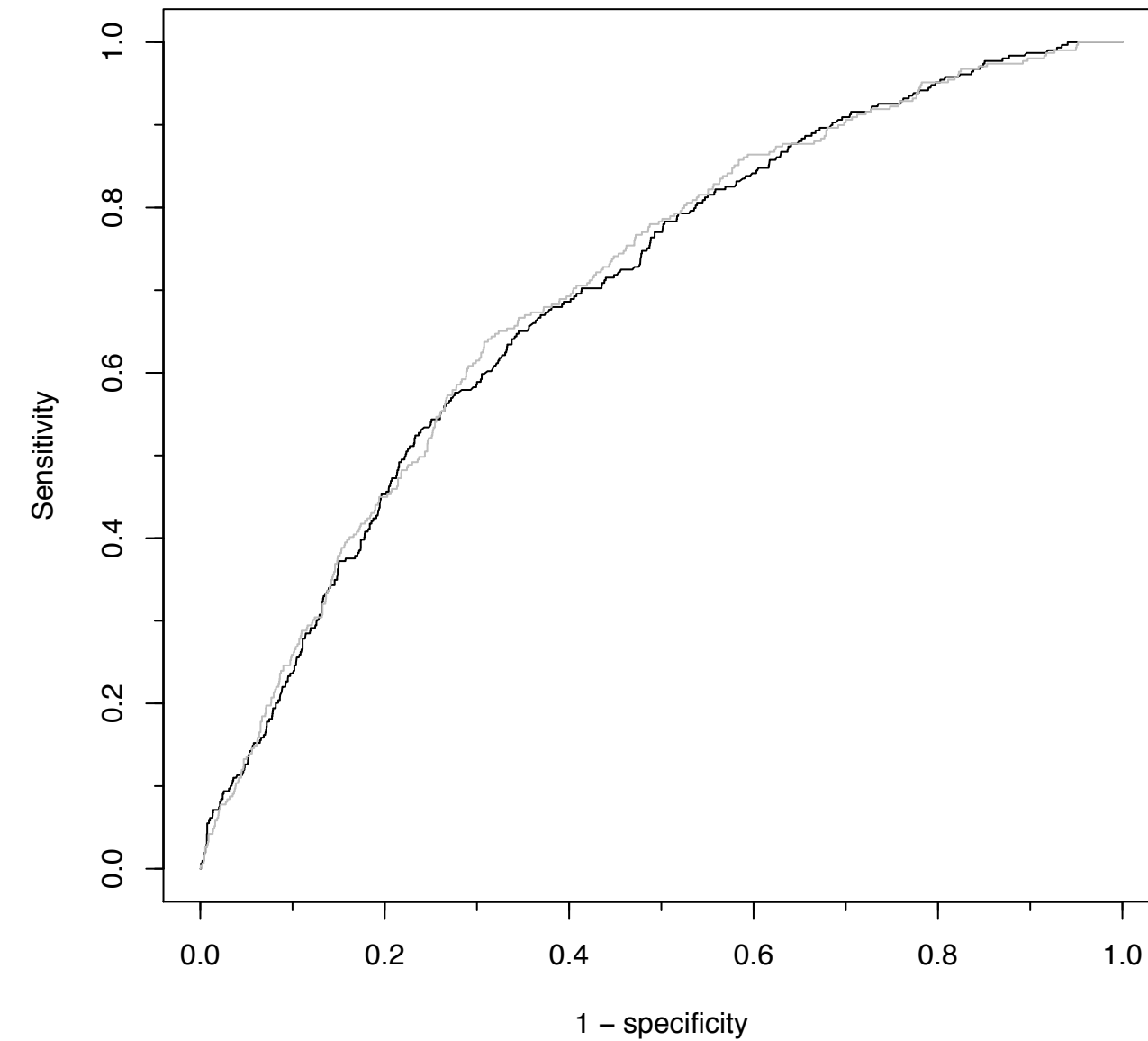**NN**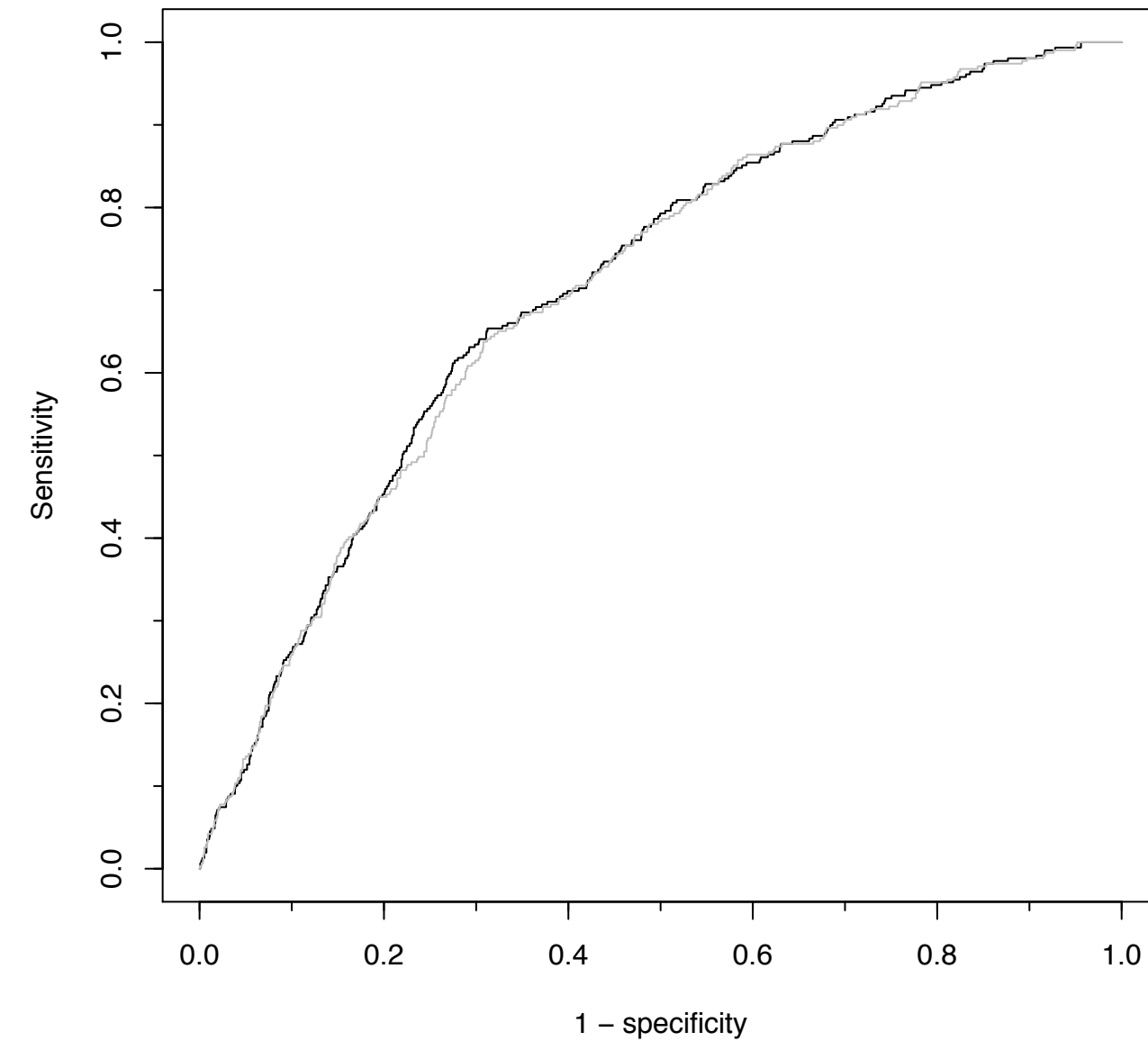

Supplement: Supplementary file 9 — Figure S6. Receiver operating characteristic curves for the prediction of LGA among primiparous women (26 weeks) using elastic net, classification trees, random forest, gradient boosting, and neural networks. (PDF 192 kb) [file 12884_2018_1971_MOESM9_ESM.pdf]

**EN**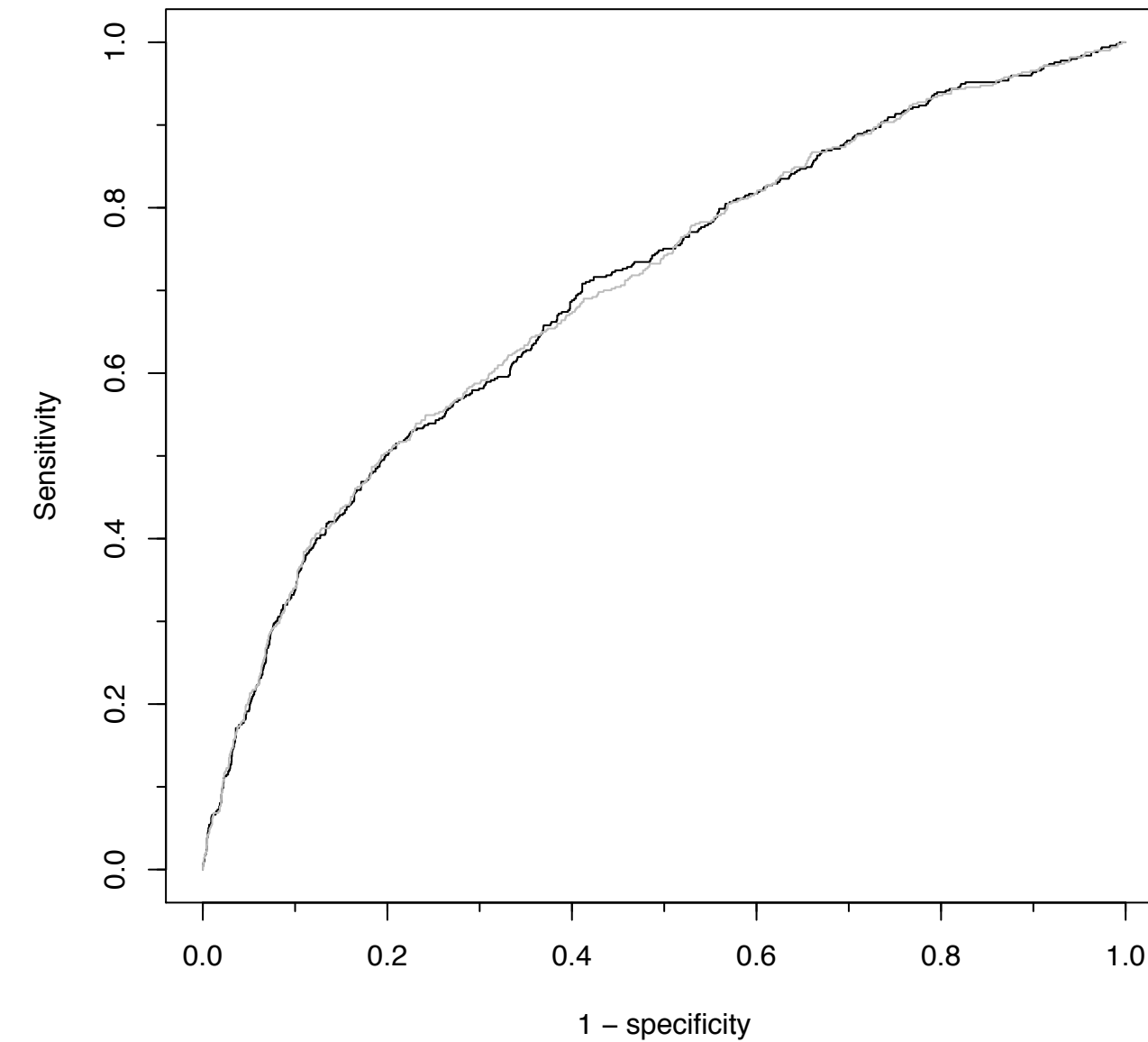**CT**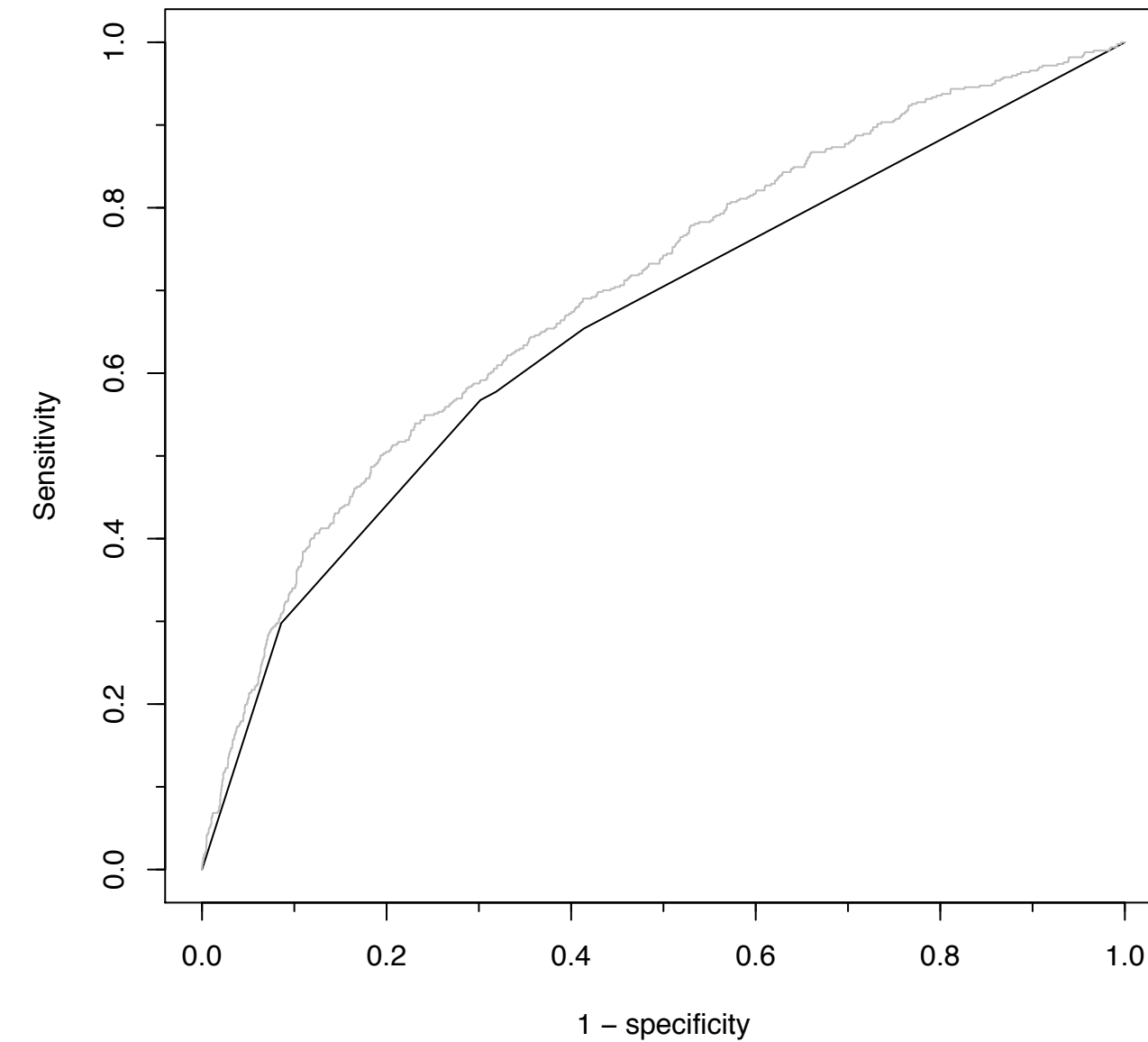**RF**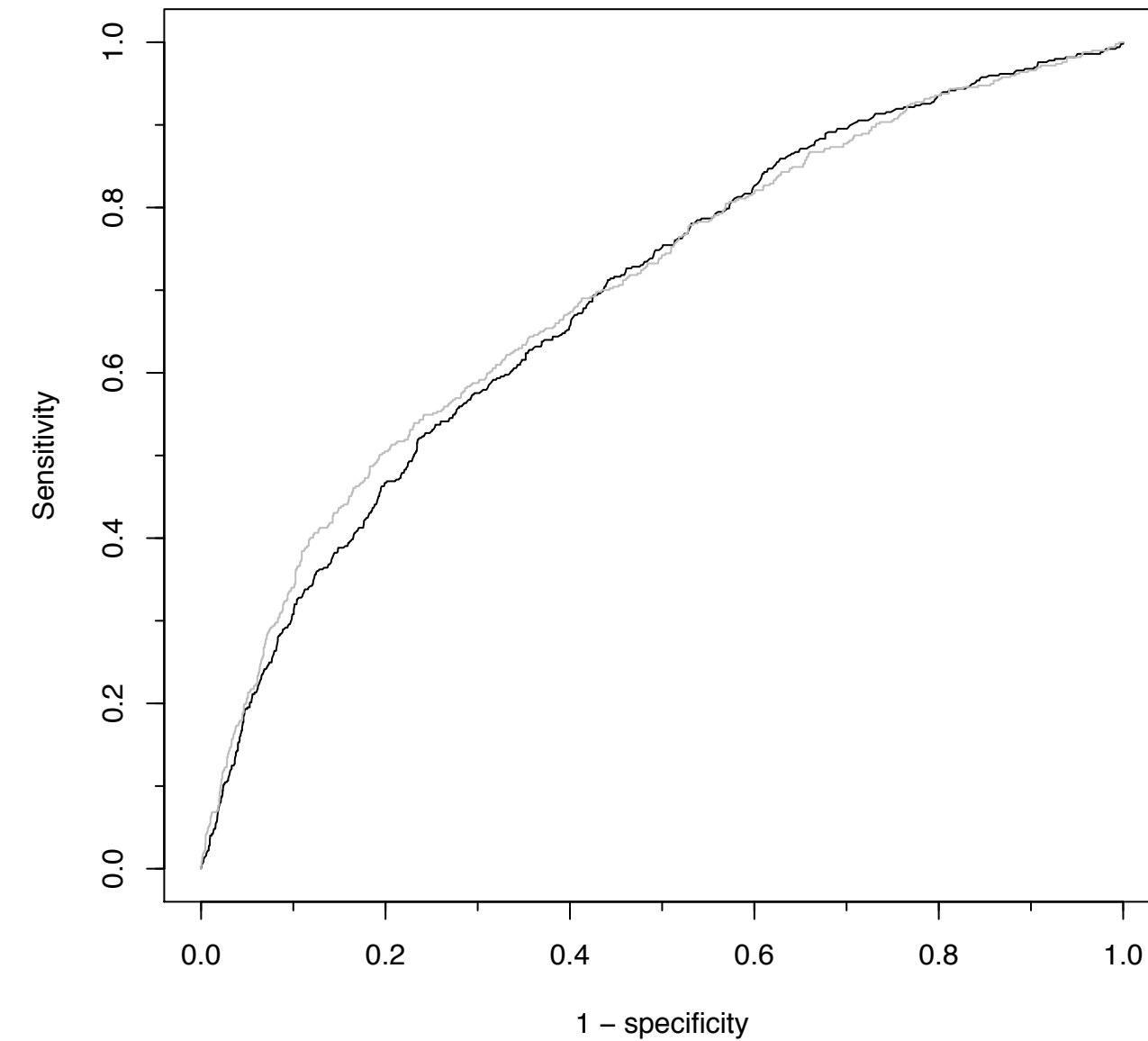**GB**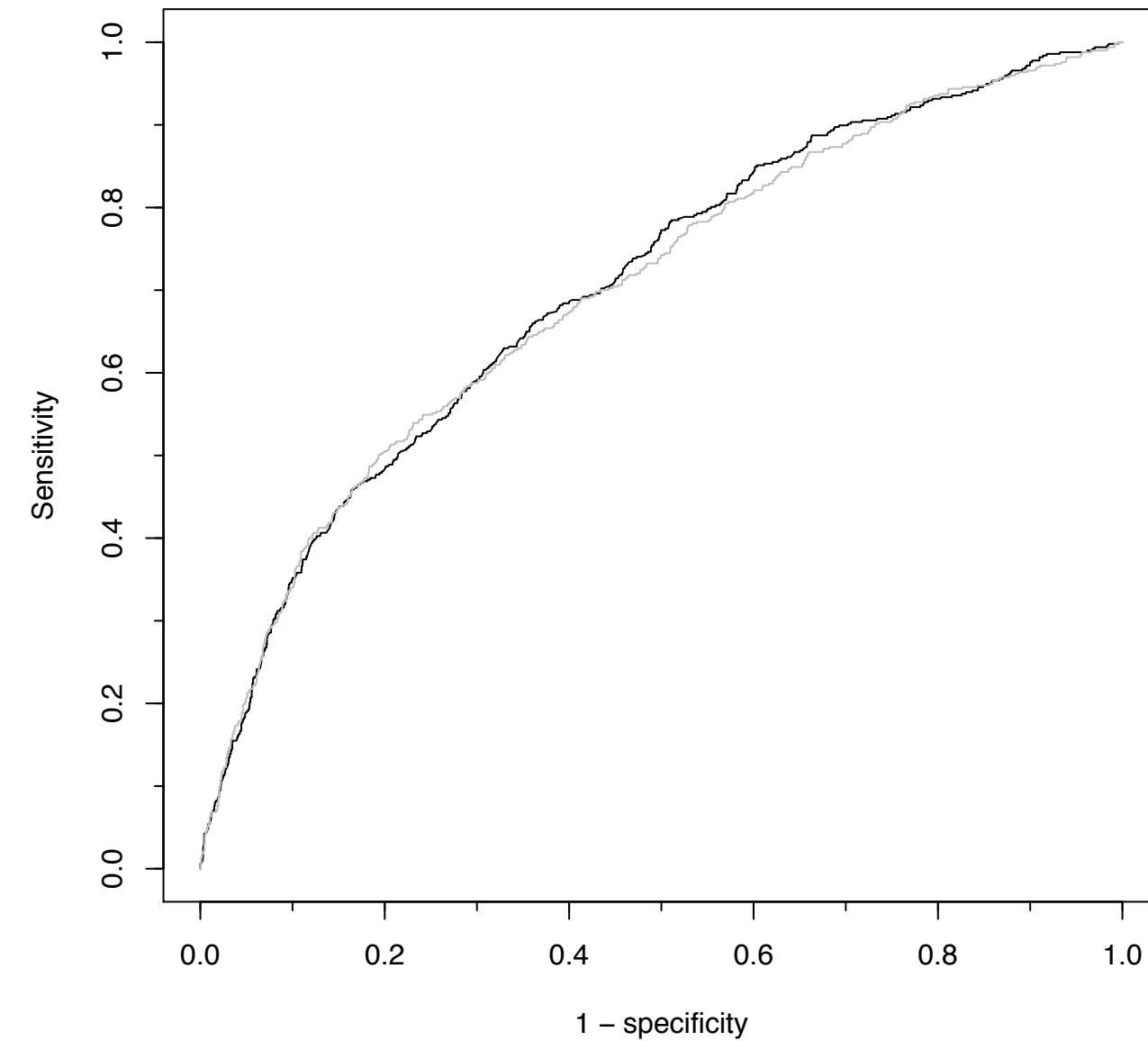**NN**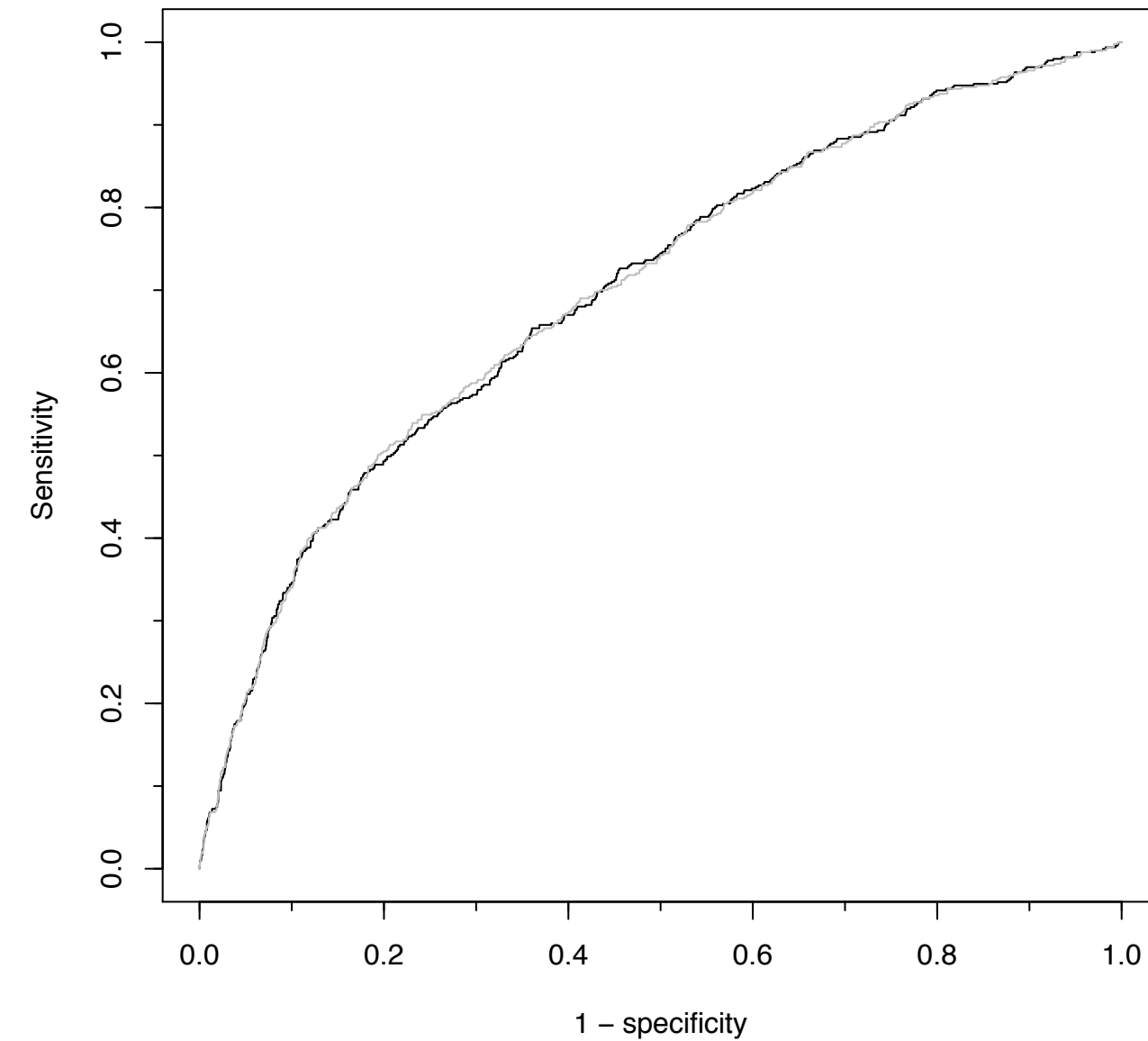

Supplement: Supplementary file 10 — Figure S7. Receiver operating characteristic curves for the prediction of LGA among multiparous women (pre-pregnancy) using elastic net, classification trees, random forest, gradient boosting, and neural networks. (PDF 200 kb) [file 12884_2018_1971_MOESM10_ESM.pdf]

**EN**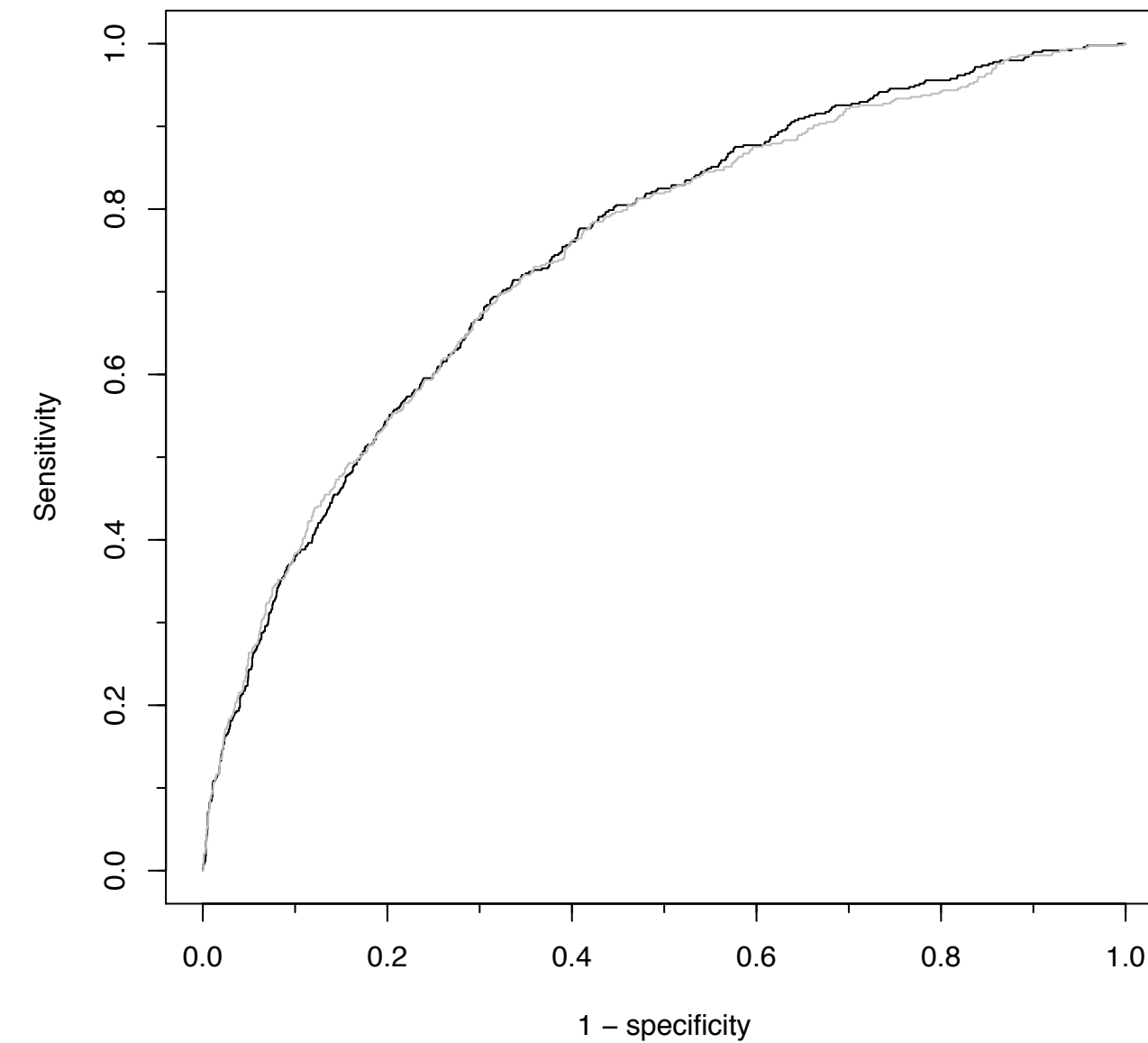**CT**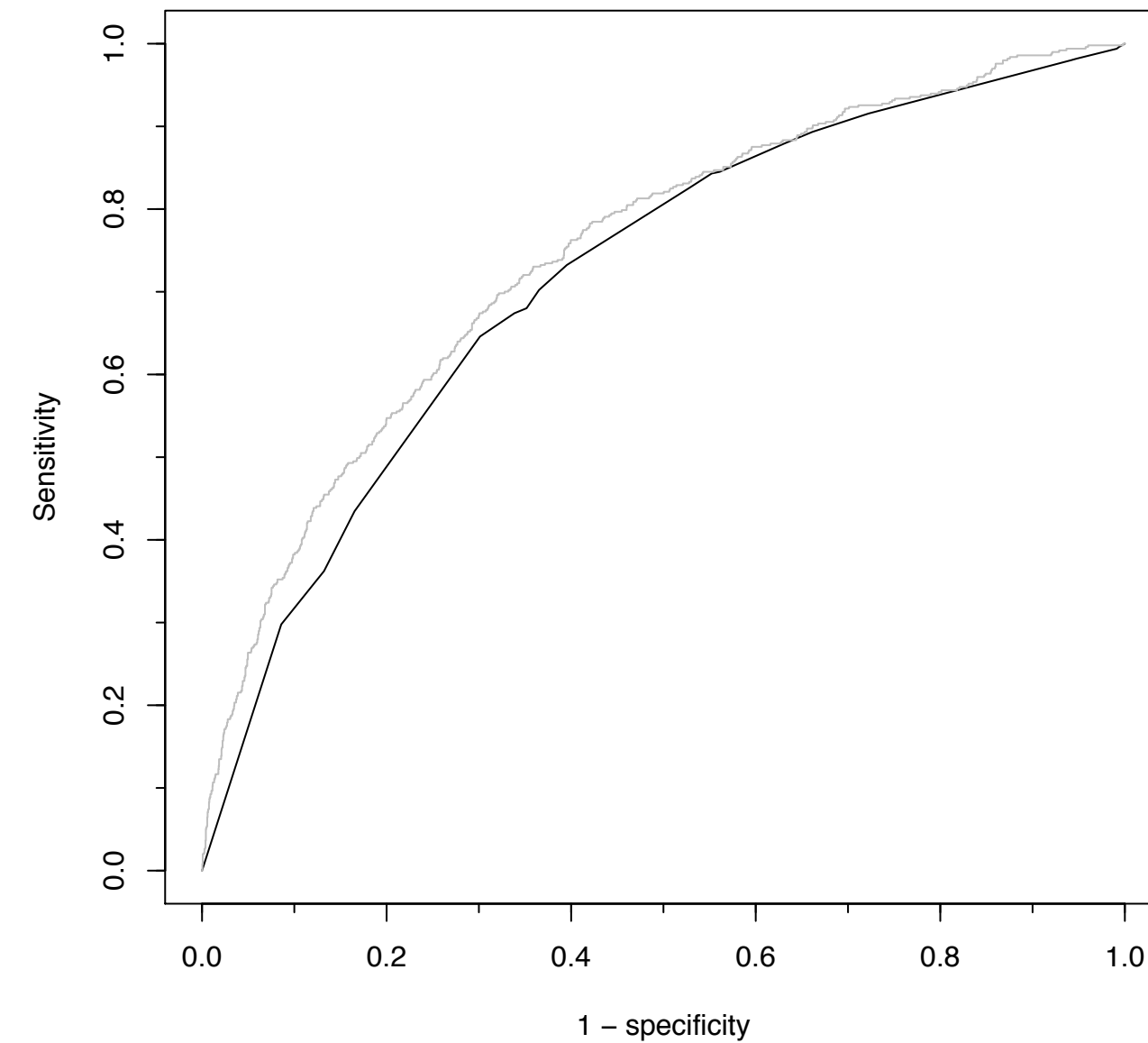**RF**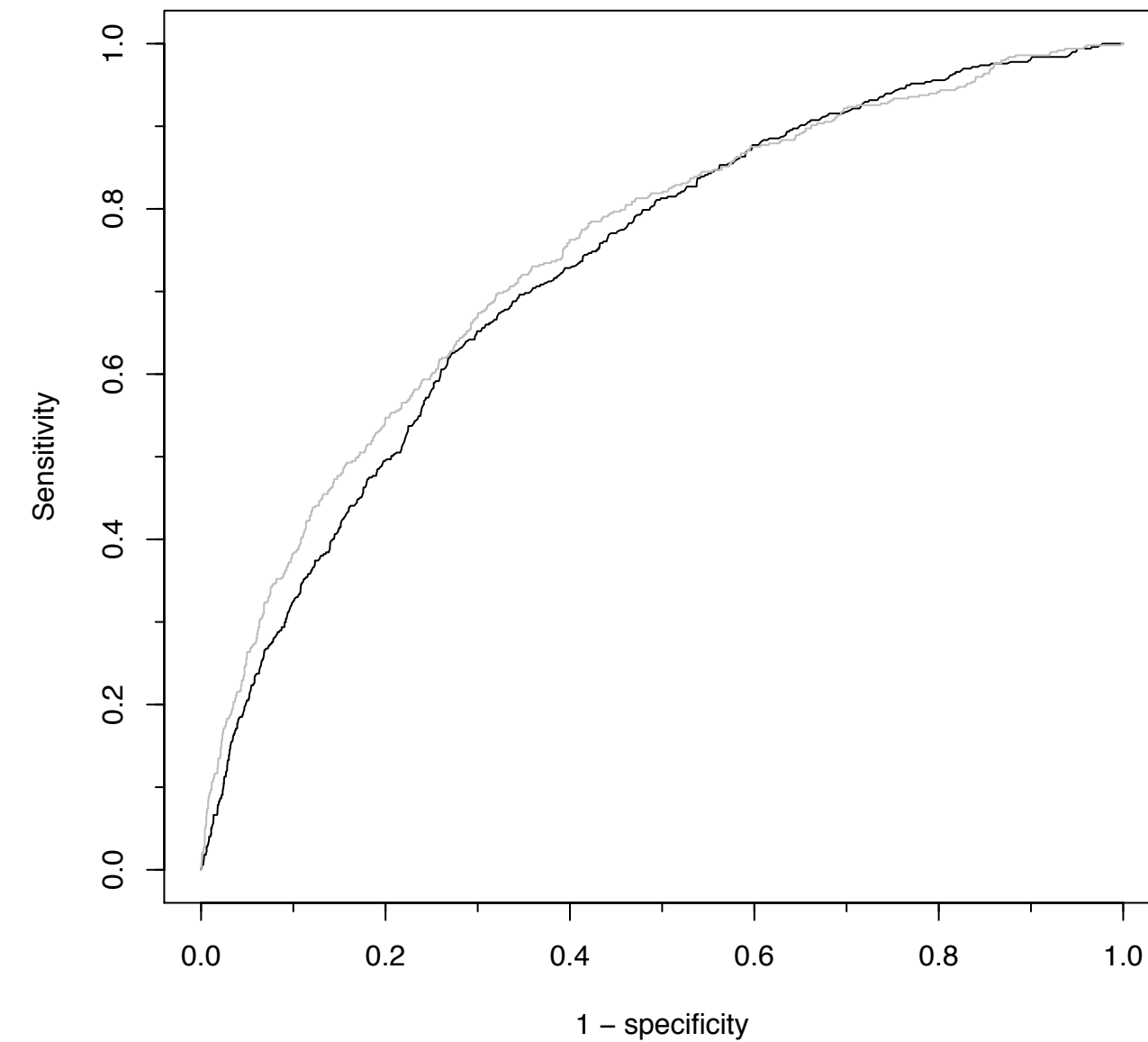**GB**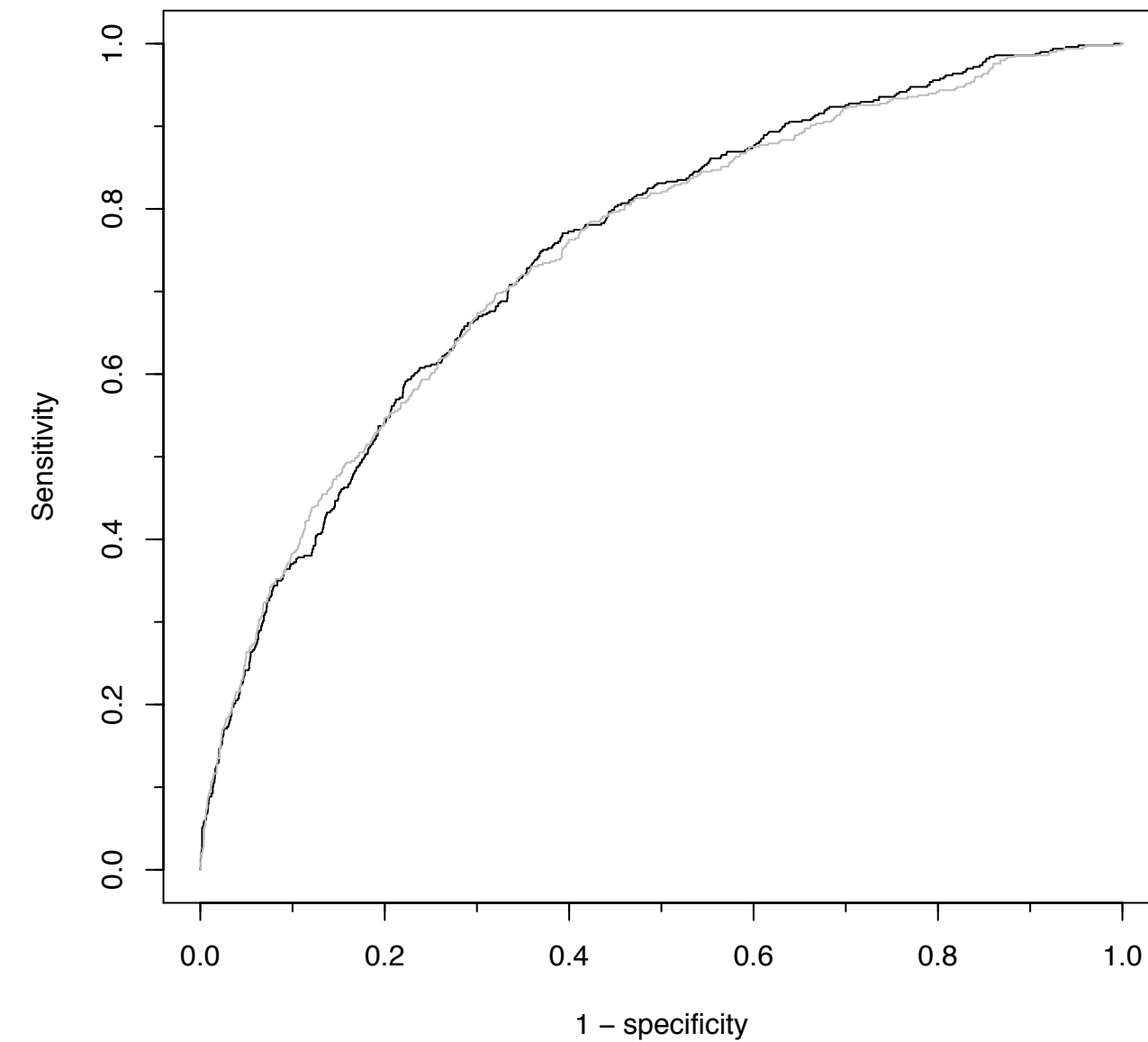**NN**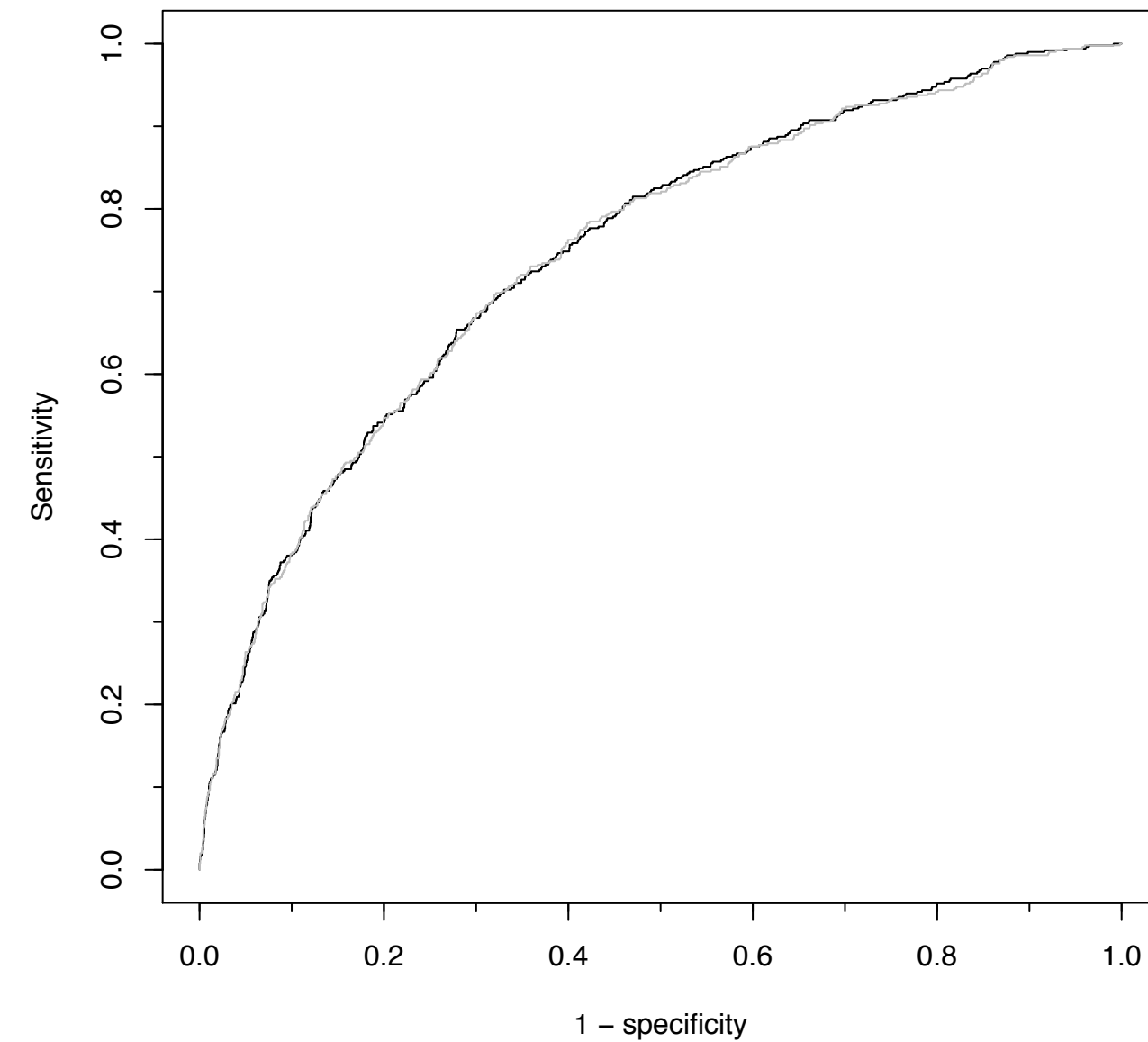

Supplement: Supplementary file 11 — Figure S8. Receiver operating characteristic curves for the prediction of LGA among multiparous women (26 weeks) using elastic net, classification trees, random forest, gradient boosting, and neural networks. (PDF 201 kb) [file 12884_2018_1971_MOESM11_ESM.pdf]
